# Current and emerging trends of inorganic, organic and eco-friendly corrosion inhibitors†

**DOI:** 10.1039/d4ra05662k

**Published:** 2024-10-08

**Authors:** Mahmoud A. Ahmed, Sherif Amin, Ashraf A. Mohamed

**Affiliations:** a Chemistry Department, Faculty of Science, Ain Shams University Cairo 11566 Egypt mahmoudmahmoud_p@sci.asu.edu.eg aamohamd@sci.asu.edu.eg; b Veolia Water Technologies Cairo 11835 Egypt

## Abstract

Effective corrosion control strategies are highly desired to reduce the fate of corrosion. One widely adopted approach is the use of corrosion inhibitors, which can significantly mitigate the detrimental effects of corrosion. This systematic review provides a thorough analysis of corrosion inhibitors, including both inorganic and organic compounds. It explores the inhibition mechanisms, highlighting the remarkable inhibitive efficiency of organic compounds attributed to the presence of heteroatoms and conjugated π-electron systems. The review presents case studies and investigations of corrosion inhibitors, shedding light on their performance and application potential. Moreover, it compares the efficacy, compatibility, and sustainability of emerging environmentally friendly corrosion inhibitors, including biopolymers from natural resources as promising candidates. The review also highlights the potential of synergistic impacts between mixed corrosion inhibitors, particularly organic/organic systems, as a viable and advantageous choice for applications in challenging processing environments. The evaluation of inhibitors is discussed, encompassing weight loss (WL) analysis, electrochemical analysis, surface analysis, and quantum mechanical calculations. The review also discusses the thermodynamics and isotherms related to corrosion inhibition, further improving the understanding of inhibitor's behavior and mechanisms. This review serves as a valuable resource for researchers, engineers, and practitioners involved in corrosion control, offering insights and future directions for effective and environmentally friendly corrosion inhibition strategies.

## Abstract 2

Effective corrosion control strategies are highly desired to reduce the fate of corrosion.

## Graphical abstract

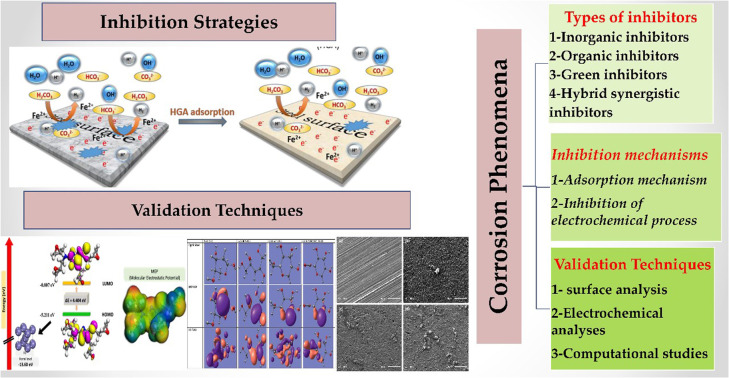

## 1. Introduction

**Fig. 1:**
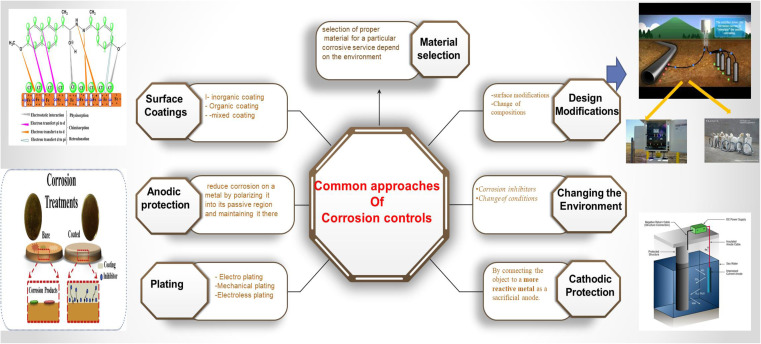
Common approaches of corrosion control.

Corrosion is a complex phenomenon that takes many forms, including uniform and localized corrosion, where atmospheric, galvanic, microbiological, pitting, crevice, erosion, intergranular, and stress-cracking corrosion types are most common.^1^ Corrosion causes irreversible degradation of materials, affecting not only the structural integrity of buildings, bridges, and infrastructures but also posing risks to human safety and life.^2,3^ In addition to the direct costs associated with replacing corroded parts and maintaining equipment, corrosion has indirect economic consequences.^4^ Corrosion-related equipment breakdowns reduce industry efficiency and cause lost productivity. Furthermore, corrosion's detrimental consequences on the environment should not be ignored. Corrosion-induced leaks and spills have the potential to pollute soil, water, and air, causing ecological disruption and posing health risks. When toxic substances from corroded materials leak into the surroundings, it can have lasting impacts on the ecosystem, necessitating extensive cleanup and mitigation activities. Because of the serious consequences of corrosion, governments and corporations all over the world are focusing their efforts on developing innovative corrosion avoidance and management approaches.^5^ While corrosion cannot be completely eradicated, several approaches can greatly reduce its incidences and consequences.^6^ These approaches often include changing the potential, surface coating, improved structural design, proper material selection, modifying the surrounding environment, and the use of corrosion inhibitors as potent protection measures, as shown in Fig. 1.^7–9^

**Fig. 2:**
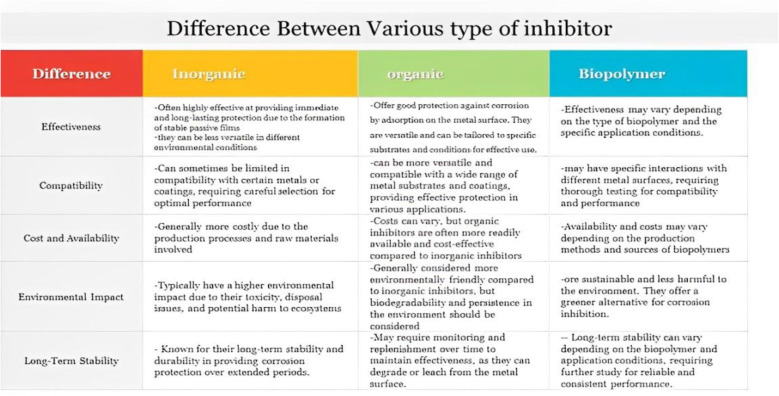
Comparison between various types of inhibitors.

Corrosion inhibitors are one of the popular approaches that have been thoroughly explored and are commonly employed for mitigating various types of uniform and localized corrosion. These inhibitors are chemical species that interact with the material's surface and/or change the characteristics of the surrounding environment to substantially boost a material's corrosion resistance in a particular environment. When applied in tiny concentrations, these inhibitors prevent or retard corrosion without substantially altering the concentration of other corrosive agents. An ideal corrosion inhibitor should be economical, simple to use, eco-friendly, and highly efficient at low dosages. Corrosion inhibitors found applications in various industrial, and pipe-line protection applications, including the oil and gas industry, cooling systems, potable water production, and the processing of metal surfaces before coating application, such as protecting reinforced concrete structures.^10,11^ Corrosion inhibitors can be classified according to their chemical composition (organic or inorganic), their oxidizing/non-oxidizing properties, or their application field (descaling, pickling, cooling water systems, acid cleaning, among others). Corrosion inhibitors stop or retard the anodic oxidation and/or cathodic reduction processes and may lead to the formation of a protective layer or film on the exposed metal surface. Thus, inhibitors may be classified as: anodic, cathodic, or mixed inhibitors. However, the most used classification scheme is that based on chemical composition. Organic inhibitors work mainly by adsorption mechanisms, whereas inorganic inhibitors typically work with electrochemical mechanisms. When it comes to performance, inorganic inhibitors have been long used since they work better over a wider temperature range and for longer periods. However, despite their relatively higher cost, organic inhibitors are thought to be safer.^12–14^ The performance of organic inhibitors relies on their composition, which typically includes various polar groups such as –OH, –OCH_3_, –COOH, –COOC_2_H_5_, –NH_2_, –CONH_2_, among others.^14–16^ These groups contain heteroatoms and non-bonding and π-electrons that enable extensive interactions.^17,18^ When these organic inhibitors contact a metallic surface, they can form adsorption layers or protective films that shield the metal from its corrosive environment and prevent its oxidation. Organic inhibitors can bind to metal surfaces by chemisorption and/or physisorption. The former involves coordinate-type bonding, *e.g.*, sharing the inhibitor's π– and non-bonding electrons with the metal d-orbitals, whereas the latter involves physical electrostatic attraction between the inhibitor and the metal surface.^13^ Inorganic inhibitors such as chromates, molybdates, phosphates, nitrites, nitrates, borates, and silicates were commonly used to combat corrosion.^19,20^ These substances interact with metal surfaces with various mechanisms that prevent corrosion. Due to cost considerations, more feasible alternatives such surface active chelates, gluconates, polyacrylates, polyphosphates, carboxylates, and phosphonates gradually replaced many of the older inhibitors. Some of the latter substances act as precipitating inhibitors that typically produce precipitates at the metal–environment interface, while others can act as passivators, scavengers, corrosion poisons, or blockers.^13,21,22^ Moreover, some old inhibitors, *e.g.*, chromates, have frequently raised environmental concerns due to detrimental impacts on soil and aquatic life.^23,24^ In response to these concerns, researchers are actively exploring environmentally friendly inhibitors as alternatives to traditional inhibitors. These eco-friendly alternatives offer numerous advantages, including ready availability of resources, non-toxicity, renewability, friendly synthesis processes, cost-effectiveness, and development of environmentally acceptable products.^24^ Some well-researched eco-friendly substitutes for harmful corrosion inhibitors include natural polymers, polysaccharides, amino acids and their derivatives, and Arabic gums.^25,26^ Green corrosion inhibitors may generally be divided into two primary groups: inorganic or organic green corrosion inhibitors. A typical example is biopolymers, which are naturally occurring compounds synthesized by cells of plants and animals, offering eco-friendly appropriate substitutes for a range of industrial uses. In contrast to synthetic polymers, they are biodegradable and do not accumulate within living organisms. Prominent examples of biopolymers encompass polypeptides, polysaccharides such as cellulose, starch, and chitosan, natural rubber, nucleic acids such as RNA and DNA, and lignin.^27^ The incorporation of heteroatoms within the complex structure of biopolymers confers enhanced adsorption capabilities and plays an integral role in corrosion inhibition.^28,29^ Consequently, extensive research has focused on exploring the anticorrosive features of biopolymers.^29^ Furthermore, corrosion inhibitors are generally added to materials such as plastics, paper, *etc.*, for metal rust protection. The process of releasing corrosion inhibitors from materials to the metal surface will also have an impact on its corrosion inhibition.^30^Fig. 2 shows comparison between various types of inhibitors.

This review delves deeply into the world of corrosion inhibitors, aiming to shed light on their various types, mechanisms, performance, and potential applications. To better understand the inhibitor's efficiency and mode of action, the review critically analyzes the numerous mechanisms involved in corrosion inhibition. Further, the review evaluates and compares the performance of various corrosion inhibitors, both organic and inorganic, through insightful case studies and investigations. Furthermore, this review highlights the use of emerging eco-friendly corrosion inhibitors derived from natural resources such as biopolymers, plant extracts, and drugs as potential corrosion inhibitors. To comprehensively evaluate a corrosion inhibitor's performance, the review highlights various analytical tools such as WL analysis, electrochemical analysis, and surface analysis. Additionally, the review discusses thermodynamics and isotherms related to corrosion inhibition, which advances our comprehension of inhibitors' behaviors and mechanisms. Furthermore, the review highlights the importance of computational studies in predicting new inhibitor's performance, thereby saving time and effort. This review bridges the gap between theory and practice, allowing researchers and practitioners to make informed decisions, develop effective corrosion inhibition strategies, and pave the way for a future in which viable inhibitors protect materials and structures from the detrimental effects of corrosion.

## 2. Corrosion inhibition mechanisms

The inhibition of metal corrosion can be achieved through one or more of the following mechanisms:

### 2.1 Adsorption mechanism of corrosion inhibition

There are multiple paths in which corrosion inhibitors can adsorb onto metal surfaces. These include strong chemisorption bonds due to chemical interactions of the metal surface and the inhibitor, as well as weak physical adsorption forces, such as hydrogen bonding, electrostatic attractions, or van der Waals interactions. Although, recent studies have shown that van der Waals interaction has little impact on identifying preferential sites compared to electrostatic interactions, it still plays a role.^31,32^ Commonly, organic inhibitor molecules comprise π-electrons and/or lone-pair electrons of heteroatoms (N, O, S, P, …), where the latter can participate in protolytic equilibria yielding positively or negatively charged moieties depending on the medium pH.^33,34^ Metallic species that experienced partial oxidation acquire positively charged surface sites that can attract negatively charged counterions like chloride and sulfate, as well as electron-pair donor moieties from inhibitors. In some cases, however, these counterions adhering to the metal surface result in negative charges that strongly interact with protonated groups of some organic inhibitors.^35^ Additionally, the inhibitor's electron-pair donor moieties can participate in coordinate bond formation with metals' low-energy empty orbitals. The adhesion forces between the substrate and the inhibitor molecules demonstrate greater potency in chemisorption than in physisorption processes. Consequently, chemisorption boasts heightened adsorption energy, thereby establishing itself as a superior approach for corrosion inhibition.^36^ Furthermore, an important aspect of some adsorption processes is referred to as retro-donation, which involves electron transfer from occupied metal orbitals to unoccupied anti-bonding orbitals of the inhibitor's heteroatoms. This retro-donation results in a synergistic chemical bonding effect.^13^ Fig. S1† depicts possible corrosion inhibition mechanisms *via* chemisorption and physisorption pathways involving organic inhibitors.^36^ Furthermore, the inhibitor's adsorbed molecules may create a protective layer on the metal surface, which might serve as a physical barrier.^37^

In another instance, XPS examination was carried out to identify the adsorption of Isatin-CS self-assembled monolayers (SAMs) on Q235 carbon steel. The physical and chemical interactions are evidenced through the presence of two types of nitrogen in the XPS data, reflecting that the Isatin-CS SAM are adsorbed onto the surface of steel *via* both mechanisms^38^ In another study, the N (403 eV)/N (398.1 eV) peak area ratio stays the same at varying inhibitor concentrations. This indicates that when the steel surface is fully coated, the proportion of molecules in different orientations is constant. Hence for nitrogen-functional inhibitors, adsorption can happen *via* electrostatic bonding between the steel surface and the N group^39^ XPS analysis results confirm the chemisorption of DAPO (2,5-bis(4-dimethylaminophenyl)-1,3,4-oxadiazole) on the MS surface. The existence of an N–Fe bond complex reflects that DAPO was chemisorbed onto MS surface, corroborating the thermodynamic findings. Furthermore, the addition of DAPO promotes the generation of a robust and insoluble oxide layer (Fe_2_O_3_, FeOOH) on the MS surface, thereby promoting its corrosion resistance.^40^ A similar study showed that the peak at 710.28 eV (N–Fe/S–Fe) also reflects that the inhibitor interacts with the metal surface to form coordination bonds.^41^

The majority of organic inhibitors adhere to the target metal surface by displacing water on the surface and creating a tight barrier, as reveled by many studies.^42^ The physical barrier prohibits corrosive substances like oxygen, water, and aggressive anions from approaching the metal surface. These barriers can affect both anodic and cathodic processes, thus slowing down both the chemical and electrochemical corrosion processes. The corrosion inhibitor's chemistry plays a crucial role in its inhibition performance. The inhibitor's specific chemical structure, functional groups, electron density, and molecular weight can all affect its ability to adsorb onto the metal surface and form a protective layer or film.^43^

To summarize, in corrosion inhibition by adsorption, the inhibitor's molecules or their ions adhere to anodic or cathodic sites, resulting in the blocking of active zones, a shift in anodic and/or cathodic potentials, and/or the formation of a protective barrier or film.^43^

For instances, the researchers examined the mechanism of *Euphorbia heterophylla* L. extract as a potent inhibitor for mild steel (MS) when exposed to a 1.5 M HCl solution and proved physical adsorption as the dominant mechanism behind the inhibition process.^44^ Furthermore, polyaspartic acid (PASP) was explored as an environmentally friendly MS corrosion inhibitor in a 3% NaCl solution.^45^ By establishing an adsorption layer on the metal surface, PASP demonstrated a moderate inhibitory efficacy of 61% at a concentration of 2.0 g L^−1^. However, at a concentration of 0.5 g L^−1^ PASP, the addition of zinc ions further increased the inhibitory efficiency to 97%, indicating a synergistic effect between PASP and Zinc ions. As shown in Fig. S2a–d,† when compared to a blank specimen, scanning electron microscopy (SEM) images verified that a protective inhibitor coating (PSAP or PSAP/Zn) was present on the MS surface. Zinc ions, in particular, produced a thicker PSAP/Zn protective layer that functioned synergistically as a cathodic inhibitor.^45^ In another study,^46^ the researchers explored the effectiveness of PASP and threonine (Thr) in preventing corrosion within simulated cooling water. The findings demonstrated that the PASP-Thr exhibited superior corrosion inhibition compared to PASP alone. This synergism can be attributed to the ability of PASP-Thr to create a protective film on the carbon steel surface, utilizing a combination of chemical and physical–chemical adsorption approaches, as shown in Fig. S2e.† The unique characteristics of PASP-Thr, such as its abundance of polar groups, and considerable molecular weight facilitate strong adherence and uniform coverage on the carbon steel surfaces.^46^

Thermodynamic information can be utilized to determine the type of adsorption exhibited by a corrosion inhibitor, whether it is chemisorption or physical adsorption. When the absolute value of Δ*G*^0^_ads_ is larger than 40 kJ mol^−1^, chemisorption takes place, signifying the creation of a chemical bond between the inhibitor and the metal surface.^47^ In contrast, physisorption happens when the absolute value of Δ*G*^0^_ads_ is lower than 20 kJ mol^−1^, suggesting an electrostatic interaction between the inhibitor's molecules and the metal surfaces.^16^ However, in many cases, the adsorption mechanism was found to be a combination of both physical and chemical interactions between the inhibitor and the metal surface.^16,47^

### 2.2 Electrochemical mechanisms of corrosion inhibition

Through an electrochemical mechanism, corrosion inhibitors smoothly suppress corrosion on metal surfaces by suppressing the anodic and/or cathodic reactions occurring during a corrosion process. This is achieved by inhibiting the cathodic reduction reaction rate and/or preventing the anodic oxidative metal dissolution.^32,48^ Corrosion inhibition employing cathodic inhibitors involves several mechanisms. Firstly, these inhibitors increase the overpotential of the cathodic reaction, making corrosion more difficult to occur. By raising the overpotential, the rate of corrosion is effectively decreased. Cathodic inhibitors, *e.g.*, polyphosphates, zinc salts and cerim(iii) salts, also work by blocking the cathodic reaction through deposition at cathodic sites, *e.g.*, through the formation of insoluble compounds, or by increasing the metal liability to hydrogen.^37,49^ This blocking action effectively hinders corrosion by lowering the availability of the cathodic reactant. The presence of cathodic inhibitors acts as a barrier, disrupting the flow of electrons and ultimately reducing the overall corrosion rate. Conversely, anodic protection takes place when a corrosion inhibitor is adsorbed on the surface and forms an oxide film on the metal surface. This protective film acts as a protective barrier, inhibiting the anodic reaction. Anodic inhibitors can be categorized into non-oxidizing ions (silicates, tungstates, and phosphates) which demand oxygen for protection, and oxidizing anions (*e.g.*, nitrites, and chromates) that can protect metals without external oxygen. Regardless of the type, anodic inhibitors may cause pitting issues and accelerated corrosion rate if concentrations are too low. Therefore, monitoring inhibitor levels is crucial.

For instance, polarization curves of MS in simulated cooling water (SCW) with various concentrations of silicate or phosphate inhibitors demonstrated that the cathodic Tafel slopes decreased, while, it was observed that an increase in the concentration of SiO_3_^2−^ or PO_4_^3−^ led to an enhancement in the values of anodic Tafel slope, with phosphate having a greater effect. This implies that both SiO_3_^2−^ or PO_4_^3−^ act as anodic inhibitors by impacting the anodic reaction of metal dissolution.^50^ Another study researched the synergized inhibition of Ce^4+^/melamine on the corrosion behavior of aluminum alloy (AA2024) in a 3.5% NaCl solution.^51^ The results showed that the corrosion current density of the sample was significantly lowered compared to the blank or single inhibitor samples. Additionally, examination of Tafel lines of polarization curves of samples in different inhibitor's concentrations showed that the anodic branches of all curves exhibited similar behavior, but with a decrease in the corrosion current density (*I*_corr_) attributable to a reduction in the cathodic current. These findings suggest that Ce^4+^/melamine solution acts as a cathodic-type inhibitor.^51^ However, in a study of N80 steel corrosion in a concentrated tetrapotassium pyrophosphate solution and its corrosion control by vanadate, the addition of 0.5 wt% NaVO_3_ caused a drop in the passive current density by 10–100, showing that NaVO_3_ functioned as an anodic inhibitor.^52^

### 2.3 Summaries of corrosion mechanisms

**Fig. 3:**
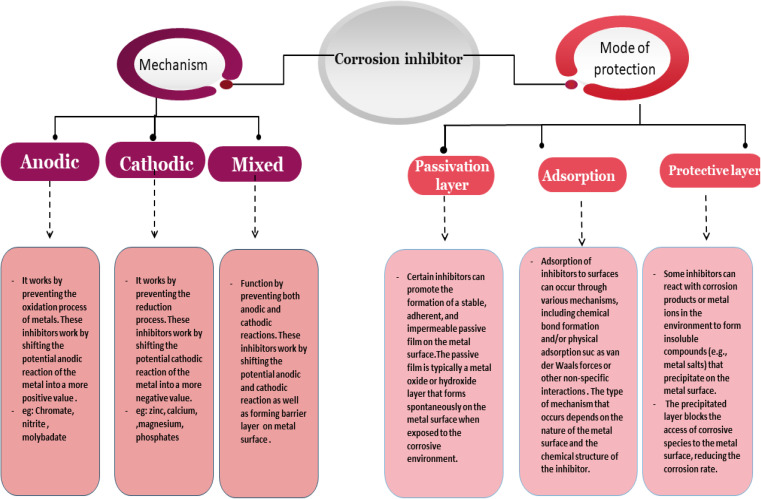
Scheme of corrosion inhibitor role.

Corrosion inhibitors can be classified into different categories based on their mechanism of action, including anodic, cathodic, and mixed inhibitors. Anodic inhibitors work by inhibiting the anodic metal dissolution reaction. Cathodic inhibitors work by inhibiting the reduction reaction, which is a crucial step in corrosion processes. Mixed inhibitors work by reducing both the anodic and cathodic reactions, leading to a more comprehensive corrosion inhibition. The synergistic interplay between the anodic and cathodic processes can result in a higher overall inhibition efficiency. Based on the mode of protection, inhibitors can form a passive layer, adsorb on the metal surface, or form a protective layer. Fig. 3 illustrates a scheme of corrosion inhibitor's role.

## 3. Type of corrosion inhibitors

In the realm of inhibition, we can differentiate between organic and inorganic inhibitors. Inorganic inhibitors exert their influence by retarding or preventing the anodic and/or the cathodic reactions of a corrosion cell. Conversely, organic inhibitors possess a multifaceted nature, displaying adsorption action, cathodic, anodic effects, or a mix of them. By classifying inhibitors into these categories, we gain a clearer understanding of their mechanisms and functionalities.

### 3.1 Inorganic inhibitors

An anodic inhibitor causes passivation by coating the metal surface with a protective layer, *e.g.*, oxide layer. Anodic or passivation inhibitors include nitrite, silicate, phosphate, chromate, and molybdate, among others. On the other hand, cathodic inhibitors operate through various approaches, such as cathodic precipitation, cathodic poisons, or oxygen scavenging. Cathodic inhibitors include salts of magnesium, calcium, zinc, and others. Table 1 lists some of the common inorganic corrosion inhibitors along with their important characteristics.

**Table 1:** Characteristics of some representative examples of inorganic corrosion inhibitors

Inhibitor	Environment	Nature of adsorption	Conc.	Method of corrosion monitoring	IE%	Ref.
Zn^2+^	Carbon steel/sea water	N. A., cathodic inhibitor	6 ppm	PDP, EIS	83.3%	49
Sodium silicate	Carbon steel/simulated cooling water	Langmuir, anodic inhibitor	1 × 10^−2^ M	PDP	74.0%	50
Sodium phosphate					95%	
Silicate–phosphate					62.3%	
Sodium vanadate	N80 steel/concentrated K_4_P_2_O_7_ solution	N. A., anodic inhibitor	0.5 wt%	WL, PDP, EIS, XPS, SEM	99.85	52
Sodium nitrite	Carbon steel/5 mM chloride	N. A., anodic inhibitor	9 mM	PDP, EIS, SEM	90%	53
Sodium nitrite	Carbon steel/simulated primary cooling water ductile cast iron/simulated primary cooling water	N. A., anodic inhibitor	1000 ppm	EIS, DFT, PDP, EIS, XPS, SEM	N. A.	54
			10 000 ppm			
Sodium nitrite	Carbon steel/250 Nacl	N. A., anodic inhibitor	175 ppm	PDP	99.061	55
Sodium nitrite	Carbon steel/simulated cooling water	N. A., anodic inhibitor	500 ppm	PDP	90%	56
Sodium nitrite	Carbon steel/tab water	N. A., anodic inhibitor	200	EIS, PDP, EIS, XPS, SEM	N. A.	57
Sodium nitrite	Carbon steel	N. A., anodic inhibitor	2 g L^−1^		65%	58
Sodium nitrite			2 g L^−1^		53%	
Nitrite + molybdate			<2 g L^−1^		93%	
Sodium molybdate	Carbon steel/0.1 M HCl	Langmuir, anodic inhibitor	300 ppm	WL, PDP	93.28	59
	0.2 M HCl				97%	
	0.3 M HCl				94.65%	
	0.4 M HCl				93.29%	
	0.5 HCl				88.99	
Sodium tungstate	Carbon steel/0.1 M HCl		300 ppm		79%	
	0.2 M HCl				86.8%	
	0.3 M HCl				83.06	
	0.4 M HCl				81.17%	
	0.5 HCl				71.87%	
Sodium molybdate	Carbon steel/Ca(OH)_2_ + H_2_SO_4_ + HNO_3_ (pH = 8)	N. A., anodic inhibitor	0.04%	EIS, PDP	80%	60
Sodium molybdate	Carbon steel/Saturated Ca(OH)_2_ + 0.5 M NaCl (pH = 12.5)	N. A., anodic inhibitor	2882 ppm	EIS, PDP, XPS	97%	61
Sodium molybdate	Carbon steel/0.01 M NaCl + 0.1 M NaHCO_3_	N. A., anodic inhibitor	2059 ppm	EIS, PDP, SEM-EDX	97.9%	62
Sodium molybdate	Carbon steel/simulated cooling water	N. A., anodic inhibitor	400 ppm	WL, SEM	98.5%	63
Sodium tungstate	Iron-based alloys/10% H_2_SO_4_ (pH < 1)	N. A., anodic inhibitor	33 × 10^3^ ppm	PDP, SEM-EDX	97%	64
Sodium tungstate	Carbon steel/1 M NaOH (pH = 14)	Langmuir, anodic inhibitor	100	WL, OCP, PDP	30.71	65
			200		45.75%	
			400		62.41%	
			800 ppm		73%	
Sodium tungstate + ZnSO_4_	Mild steel/natural seawater	N. A., mixed type inhibitor	1000 ppm + 300 ppm ZnSO_4_	WL, EIS, PDP, XPS	84.81	66
Sodium nitrite sodium silicate	Carbon steel/3% NaCl	N. A., anodic inhibitor	0.5%	WL	94.1%	67
			2%		92.5%	
Sodium silicate	Carbon steel/3.5% NaCl	Langmuir/anodic inhibitor	1250 ppm	WL, EIS, PDP,SEM	92%	68

#### 3.1.1. Nitrite inhibitors

Nitrite, a widely studied inorganic inhibitor, has received considerable attention in scientific literature owing to its affordability and effective inhibition features.^69,70^ Nitrite inhibitors tend to minimize the corrosion current density and move *E*_corr_ to a noble direction.^54^ For instance, potentiodynamic polarization (PDP) curves were employed to examine how the addition of NaNO_2_ impacts the corrosion inhibition of carbon steel in de-aerated SCW.^54^ When NaNO_2_ was absent, the metal easily corroded; however, when NaNO_2_ was introduced at levels of 10 and 100 ppm, carbon steel exhibited a transition from active to passive behavior. Remarkably, carbon steel displayed powerful passivation features when NaNO_2_ was added at a level of 1000 ppm.^54^ The mechanism behind nitrite's inhibitory action has been the subject of investigation by numerous researchers.^53,71^ Specifically, in the case of iron, it has been proposed that nitrite ion serves as an oxidizing agent, facilitating the conversion of ferrous to ferric ions and thus playing a key role in the formation of a sturdy and enduring oxide passive film on steel surfaces.^37^ The resulting protective film was primarily composed of magnetite in both of the outer and inner layers.^53^ The environmental parameters including the metal or alloy type and composition, medium pH, aggressive ion type and concentration, and the temperature have a great impact on the performance of nitrite inhibitor.^55,71–73^ For instance, it was shown that when chloride ions exist at great levels, while using insufficient amounts of nitrite promoted the likelihood of encountering pitting phenomena owing to the proximity of pitting potential (*E*_pit_) to the open circuit potential (OCP).^71^ Furthermore, the nitrite was found to be more resistant than chromate to the damage caused by chloride ions; however, it was of marginally less resistant to the effects of sulfate ions.^74^ Moreover, when the nitrite level was equal to or less than 53 mmol L^−1^, it was demonstrated that the sulfate-generated corrosion was more likely to occur than that of chloride.^75^ Furthermore, in SCW solution containing chloride ions, the effect of nitrite ions on MS corrosion was examined and demonstrated that in alkaline and near-neutral media (pH 6 and above), nitrite ions efficiently inhibited steel corrosion; however, in acidic environments (pH 4 and below), nitrite ions promoted corrosion.^56^ A prolonged soaking period, up to 24 hours, enhanced the inhibitory effect of NaNO_2_ afterward, for all nitrite levels at and above pH 6, it stayed comparatively steady.^56^ Additionally, through PDP curves' analysis, the researchers found that the addition of nitrite to de-aerated tap water containing carbon steel, caused the corrosion potential to move toward the noble region, resulting in a minimized corrosion rate (Fig. S3a†).^57^ SEM examination unveiled that the nitrite inhibitor facilitated the creation of a γ-Fe_2_O_3_ passive film at nitrite levels of 100–200 ppm, while lower nitrite levels of ≤50 ppm led to the formation of corrosion products owing to uniform corrosion (Fig. S3c and d†).^57^ In terms of electrochemical impedance spectroscopic (EIS) studies, it was observed that the carbon steel with <100 ppm nitrite exhibited a higher vulnerability to localized corrosion due to its smaller anode area and larger cathode area, whereas a submerged solution containing <50 ppm nitrite primarily experienced uniform corrosion. In another study, the impact of temperature on the ability of nitrite to inhibit corrosion was examined and the researchers found that as the temperature of the medium increased, *I*_corr_ increased, while *E*_corr_ was shifted towards more negative values.^53^ It became evident that both βc and βa increased with temperature, but the increase in βa was significantly greater than that of βc, revealing that higher temperatures generally promote the dissolution of metal ions by enhancing the activation of the tested metal surface, resulting in a minimization in the resistance of the oxide film existing on the metal surface. In this respect, the primary focus has been placed on regulating the level of nitrite within an optimal range to achieve superior inhibitory characteristics and consequently mitigate the likelihood of pitting corrosion. In addition, nitrite has been employed collaboratively with other inhibitors, such as molybdate, to mitigate mild-steel and carbon-steel corrosion in chloride solutions lacking ample oxygen.^58,76,77^

#### 3.1.2. Molybdate inhibitors

Due to its low toxicity and environmental friendliness, the molybdate ion has been extensively examined as a corrosion inhibitor for a variety of metals and alloys in a range of adverse environments.^78–80^ The first exploration of the inhibitory impact of molybdate on carbon steel corrosion in a neutral medium was carried out in 1953 (ref. 81) and its inhibition mechanism was ascertained in 1955.^82^ The researchers found that although molybdate was less effective in de-aerated water, it demonstrated high performance in aerated water. Subsequently, numerous studies have explored the application of molybdate as a powerful inhibitor for steel corrosion.^59,83^ Molybdate falls under the category of oxidizing anodic inhibitors, requiring the existence of external oxygen to generate a protective oxide layer on iron alloys. As the anode undergoes a chemical reaction, ferrous ions are produced (Fe^0^ → Fe^2+^ + 2e^−^). These ferrous ions then interact with molybdate ions, resulting in the generation of a molybdate–ferrous complex that does not provide adequate protection.^84^ Subsequently, this complex undergoes oxidation when it comes into contact with dissolved oxygen to yield a thin protective and insoluble molybdate–ferric complex, which combines with ferric oxide to provide an enhanced corrosion protection.^85^ Thus, a molybdate thin layer on the surface of MS considerably impeded localized corrosion in NaCl solutions.^86^ This protective molybdate film demonstrated a dual-layer structure: the outer layer primarily contained Fe_2_(MoO_4_)_3_, while the inner layer consisted of ferric hydroxide/oxide.^76,77^ In another investigation, the researchers found that MoO_2_, MoO_4_^2−^, and MoO_3_ made up the protective coating of carbon steel that developed in a solution containing 0.5 M NaHCO_3_, 0.5 M NaCO_3_, and 1.5 M NaCl.^87^ Interestingly, it was demonstrated that the MoO_4_^2−^ peak had a greater intensity than any other species in the protective layer. Additionally, after being subjected to the molybdate solution, MS specimens were examined employing Auger Electron Spectroscopy (AES). The findings demonstrated that a 3 nm molybdenum oxide layer was discovered next to a 6 nm layer of iron oxide.^88^ Furthermore, molybdate is thought to fortify the topmost layer of hydrated iron oxide by establishing hydrogen bonds with hydroxide groups present on the surface. This reinforcement imparts a negative surface charge, effectively preventing aggressive anions such as chloride and sulfate from approaching the metal surface and inhibiting the departure of ferrous ions from the metal.^85,87^ Molybdate aids in slowing down pits formation by releasing adsorbed molybdate when a breach in the protective layer arises as evidenced by shifts of the pitting potential (*E*_pit_) to the positive direction.^87^ This molybdate then precipitates as complexed iron-molybdates within the tiny pit, preventing further corrosion propagation.^85,89^ The effective range of molybdate levels is wide because it is significantly affected by the presence of high levels of aggressive anions. For instance, the researchers reported that in deionized or low-electrolyte water, a 70 ppm molybdate was an effective inhibitor; however, in the presence of 200 ppm chloride, a 466 ppm molybdate was necessary to achieve corrosion inhibition.^85^ Similarly, other researchers found that when the chloride levels was around 200 ppm, a minimum of 1000 ppm molybdate was required to provide adequate protection; however, at lower levels of 70 ppm Na_2_SO_4_ and 30 ppm NaCl, effective inhibition can be achieved with only 200 ppm Na_2_MoO_4_.^90^

#### 3.1.3. Silicate inhibitors

Alkali silicates are water soluble, nonflammable, non-toxic, and exhibit a prolonged shelf-life. There are many commercially available grades of sodium silicates (SS), which may be identified by their SiO_2_/Na_2_O weight ratio. These ratios commonly fall between 1.00 : 2.00 to 3.75 : 1.00, where grades with ratios less than 2.85 : 1.0 are termed alkaline, while those having higher ratios are neutral.^20^ Silicate inhibitors are a popular category of inorganic substances utilized to mitigate corrosion. These inhibitors function by creating a protective silicate coating on the metal surface, which effectively shields it from the harmful effects of the corrosive surroundings.^20^ However, certain studies reported that an iron oxide layer with a thin film of γ-Fe_2_O_3_ (ref. 85 and 91) or a structured Fe_2_O_3_/FeO/Fe^20^ arrangement is responsible for the protective action of sodium silicate. A possible pathway for silicate protective film development includes the displacement of water at the interface between steel and the surrounding solution, where silicates are adsorbed onto the target metal surface, resulting in a reduction in the film capacitance.^20^ X-ray photoelectron spectroscopic (XPS) analysis of the silicate protective layers on steel revealed that silicon makes up the majority of the outer film's composition that can be formed by (1) diffusion of silicate ions to steel surface with subsequent interaction with Fe^3+^, Fe^2+^, and OH^−^ ions to form insoluble silicate compounds that settle on steel surfaces and/or (2) silica hydrolysis on steel surface and formation of a protective mixed layer made of hydrated silica gel and iron oxides.^92^ Furthermore, it has been proposed that silica that is continually added to the system has self-healing qualities in addition to creating a protective coating.^93–95^ This implies that if film damage occurs (for example, from erosion or corrosion), the silicate found in the surrounding medium slowly creates a new film to heal the protective layer where the damage was caused. The generation of the healing protective film by sodium silicate inhibitor might occur gradually, over several weeks.^20,85^

#### 3.1.4. Tungstate inhibitors

Sodium tungstate has proved as a promising corrosion inhibitor in various industries for easy application and integration into various corrosion prevention methods, such as immersion, spraying, or dipping.^96^ Its unique characteristics make it a competent option for protecting various metals, *e.g.*, steel, aluminum, copper, and nickel, from corrosion in different environments.^96–98^ Moreover, tungstate exhibits favorable performance in both acidic and alkaline environments. It possesses excellent chemical stability, enabling it to resist deterioration and maintain its protective properties in highly corrosive media. This feature makes sodium tungstate particularly useful in industries where corrosive agents vary or fluctuate, providing consistent corrosion protection regardless of the pH conditions. Additionally, sodium tungstate is known for its inhibiting effects on localized corrosion mechanisms such as pitting and crevice corrosion. These types of corrosion can occur in specific areas where the protective oxide layer is compromised, leading to severe damage if left unmitigated.^96^ Furthermore, the good thermal stability of tungstate's make it a suitable choice for applications in high-temperature environments, where many corrosion inhibitors can lose their effectiveness.^96^ When tungstate is introduced into a corrosive environment, it tends to undergo polymerization that results in the generation of polytungsten anions like [W_7_O_24_]^6−^ and [W_10_O_32_]^4−^ along with the parent monomeric form WO_4_^2−^.^96^ The corrosive anions compete with tungstate-based anions to adsorb at the corroding interface of the metal sample. The existence of tungstate's lowers the adsorption of corrosive ions on the target surface and simultaneously generates an insoluble protective tungstate layer by binding to metal ions, and effectively hindering the attack of corrosive anions.^99^ Thus, tungstate's can effectively fill in gaps and fix any flaws in the protective layer.^64^ Just like MoO_4_^2−^ inhibitors, tungstate is also considered risky as it can boost corrosion when not applied in an optimum dosage. Moreover, instances of failures have been reported at high tungstate levels, under unique conditions.^100^

#### 3.1.5. Phosphate inhibitors

Phosphates are widely used as steel corrosion inhibitor in concrete protection, and boiler's water, potable water, and cooling water systems, as well as other water distribution systems.^101–103^ Thus, sodium hydrogen phosphate, disodium hydrogen phosphate, sodium phosphate, polyphosphate, and monofluorophosphate, have been used successfully in various applications. Phosphates are classified as non-oxidative anodic inhibitors that are usually effective when oxygen is present.^104–106^ Numerous investigations have been conducted to comprehend the corrosion inhibitory role of phosphates on the iron surfaces within diverse environments.^102,107^ Generally, it is widely acknowledged that the protective layer generated on the iron surface in alkaline conditions consists of a dual component structure: an internal section comprising iron oxides such as Fe_3_O_4_ or/and (Fe_2_O_3_) and an exterior section comprising various iron phosphate complexes, *e.g.*, Fe_3_(PO_4_)_2_, FeHPO_4_, and FePO_4_, where various oxidation states of iron and various protolytic equilibrated forms of phosphate are involved depending on the levels of dissolved oxygen and the medium pH.^108–110^ Various analytical techniques, including AES and XPS analysis,^111^ ellipsometric,^112^ Raman spectra,^113^ and XRF,^104^ have verified the existence of phosphate inside the outer layer's structure. Nevertheless, the features of this protective film are contingent upon various elements, namely the temperature and pH of the medium, alongside the type and concentration of electrolyte ions.^111,112,114^ Phosphates, however, may also serve as cathodic corrosion-inhibitors, under certain circumstances. In these situations, a film forms on cathodic sites, probably due to a reaction between the metal and phosphate ions in a medium.^105^ The degree of inhibition is determined by how well the film works, particularly how well it serves as a barrier. Furthermore, it was found that the inhibitory effects of polyphosphate are further promoted when divalent Ca^2+^ or Zn^2+^ cations are found in the solution.^115^ This phenomenon is associated with the formation of (1) a shield on the cathodic areas, thereby raising its overpotential, and/or (2) a shield on the anodic areas by preventing oxygen from reaching the anodic sites, thereby inhibiting corrosion.^115^ Thus, when iron was placed in a dilute orthophosphate medium containing Ca^2+^, the cathodic process was impeded by the formation of a sparingly soluble calcium phosphate film, which covered the iron surface limiting its accessibility and reaction with dissolved oxygen.^116^ Additionally, Na_3_PO_4_ solution inhibited the cathodic reduction reaction of oxygen for iron corrosion in tap water medium, probably due to the cathodic adhesion of positively charged magnesium and calcium with phosphate anions and raining the cathodic overpotential. Interestingly, the formation of these sparingly soluble particles and their subsequent adhesion to the tested metal surfaces is independent of the oxygen content of the medium.^117^

#### 3.1.6. Zinc ion inhibitor

In aqueous environments, zinc salts that dissolve in water are commonly employed as inhibitors to minimize corrosion.^118^ These salts function by forming insoluble metal hydroxides on the surface of the targeted metal. This chemical reaction takes place when the zinc salts come into contact with OH^−^ formed during the cathodic reduction of O_2_ molecules. When combined with other inhibitors like organic and inorganic anions, such as molybdate, phosphonates, and other polymeric species, zinc salts can offer promoted defense against corrosion. However, there is a growing inclination to minimize the employment of zinc-based inhibitors. This is driven by concerns regarding their environmental impact and the potential risks associated with the presence of biogenically produced H_2_S in the water, as well as water contamination caused by substances containing sulfide. These factors can result in a significant drop in the level of zinc ions, thereby compromising the performance of the inhibitor.

#### 3.1.7. Summary of inorganic inhibitors

Inorganic inhibitors perform an essential role in protecting metals in harsh circumstances. Their corrosion resistance, nonvolatility, and thermal stability make them preferred over organic inhibitors in certain applications. Understanding the impact of these inhibitors on the cathodic and anodic polarization branches are keys to predicting their corrosion prevention performance. By altering the reactions in each branch, inhibitors can either impede or prevent the corrosion process, making them valuable tools for safeguarding metals. There are three main forms of inorganic inhibitors: anodic, cathodic, and mixed inhibitors. However, environmental concerns and the high concentrations often required hinder their application. Further, the utilization of anodic inhibitors at low levels can induce the stimulation of corrosion, notably the formation of pits, constituting a substantial hazard. Considering these factors, the selection and utilization of inorganic inhibitors require careful assessment and consideration.

### 3.2 Organic inhibitors

#### 3.2.1. Organic phosphonate

Phosphonates are widely recognized and commonly employed organic inhibitors for water systems to minimize corrosion. These include compounds like HEDP (1-hydroxyethane-1,1-diphosphonic acid), AMP (amino trimethylene phosphonic acid), ADMP (amino-di(methylenephosphonic) acid), ATMP (amino-tris(methylene-phosphonic)acid), EDTP (ethylendiamine-*N*,*N*,*N*′,*N*′-tetrakis(methylenephosphonic)acid), HMDTP (hexaethylenediamine-*N*,*N*,*N*′,*N*′-tetrakis(methylenephosphonic)acid), PBTC (2-phosphonobutane-1,2,4-tricarboxylic acid), and HPAA (hydroxyphosphono-acetic acid), along with the metal cation complexes they form.^119^ Their exceptional stability, owing to strong carbon–phosphorus bonds, allows them to withstand even the harshest conditions. The activity of inhibitors containing phosphonic acid groups is primarily attributed to their ability to attach to oxidized metal surfaces through –P–O–M bonds (where M stands for metals).^120^ This assertion has been supported by various surface analytical techniques.^22,121^ For example, the impacts of HEDP to minimize steel corrosion in a soft water medium was investigated.^122^ According to the EIS measurements, there is a lowering in capacitance as the level of HEDP rises, which might be caused by the integration of organic phosphorous into the surface. The maximum inhibition of 62.59% was observed at concentrations of 25 ppm HEDP. Nevertheless, as the HEDP level rises to greater levels, a negative impact on inhibition was shown. This is because as the HEDP level rises, soluble complexes with ferrous ions are generated rather than the protective layer covering the surface. Furthermore, an ideal phosphonate inhibitor of the “complexing type” should have various features including the ability to create dense and structurally robust thin films of metal phosphonate on the surface, and should not form highly soluble metal complexes that remain soluble in the bulk without depositing onto the metal surface.^123^ If the generated film is uneven or porous, it could create localized oxygen permeation sites, leading to potential pitting of the metal surface.^123^ The performance of phosphonate-based inhibitor systems can be boosted through the existence of metal cations in solution. Thus, the researchers have extensively studied the synergistic effects of HEDP and calcium ions.^124^ Their findings reflected that the optimal anticorrosion impact is achieved when the molar ratio of Ca : HEDP was 1 : 1 under neutral conditions. Subsequent studies on MS revealed that larger Ca/HEDP solution concentrations induce the inhibitor film to develop thicker and the oxide portion in the surface layer to rise.^123,125^ In isolation, HEDP functions as an anodic corrosion inhibitor, impeding the dissolving of metals. But when paired with calcium ions, it suppresses the cathodic O_2_ reduction and lowers the anodic current. As a consequence, both components lessen the corrosion current.^125,126^ Furthermore, a protective layer on steel surface was formed by a synergistic combination of zinc ions and imino dimethyl phosphonic acid (IDMPA) inhibitor, where the formed [Zn(ii)–IDMPA] complex spread onto steel surface and combined with the generated Fe^2+/^Fe^3+^ at the anodic sites to form another protective complex proposed as [Fe(ii)/Fe(iii)/Zn(ii)–IDMPA].^127^ In the meantime, the bulk solution's free Zn^2+^ ions combine with OH^−^ ions generated at the cathodic regions, resulting in the generation of Zn(OH)_2_ precipitate. Further, the combination of zinc ions and propyl phosphonic acid (PPA) has demonstrated performance inhibition for steel at pH 6–8, where a protective film of [Fe(iii)Fe(ii)Zn(ii)-PPA], Zn(OH)_2_, and iron oxides/hydroxides was generated on steel surfaces.^128^ This film minimized the double-layer capacitance and promoted resistance to charge transfer, resulting in enhanced anti-corrosion features.^128^ It is worthy to note that, the molar ratio of zinc to phosphonate is crucial in determining the composition of the complexes responsible for film formation and inhibition effectiveness. Additionally, the levels of phosphonate and zinc individually impact the anti-corrosion effect, with higher zinc levels enhancing inhibition activity. The formed layers impede both of the iron anodic dissolution and the cathodic oxygen reduction processes.

#### 3.2.2. Heterocyclic inhibitors

Metallic corrosion can be retarded or inhibited by a variety of heterocyclic compounds. These inhibitors are organic substances that have a ring structure containing one or more heteroatoms (P, S, N, or O). Because heteroatoms are part of their structure, heterocyclic inhibitors may interact with metal ions more easily to develop stable, insoluble complexes that upon adsorption on metallic surfaces can prevent corrosion. The presence of lone pair electrons on heteroatoms enables them to readily interact with metal and results in powerful chemical adsorption. There is a well-known empirical rule regarding the inhibition performance of molecules including heteroatoms, ordering the sequence of increasing efficacy as O < N < S < P, revealing that the atoms with lower electronegativity exhibit great charge transfer and great hindering effectiveness.^43,129^ Additionally, when heteroatoms are subjected to alkaline or acidic environments, they can participate in protolytic equilibria to acquire negative or positive charges *via* deprotonation or protonation processes. Through reverse donation, *e.g.*, dπ–pπ, the heteroatom (pπ-orbital) can accept electrons from the metal (dπ-orbital). On the other hand, if the heteroatom is negatively charged, it contributes electrons to the metal surface. Powerful adsorption is advantageous for inhibition in both scenarios. It should be mentioned that heterocycles including sulfur are preferable for hindering corrosion in sulfuric acid, whereas heterocycles including nitrogen have proven to be more efficient as inhibitors in hydrochloric acid media. Still, it is thought that heterocycles with both nitrogen and sulfur atoms are considerably more potent. Numerous variables, such as the temperature, inhibitor concentration, pH, and the metal surface's features, impact the adsorption phenomena. Heterocyclic corrosion inhibitors of various kinds have been thoroughly investigated and applied to prevent corrosion. However, ring strain causes four- or three-membered heterocycles to be lower in stability than six- or five-membered rings, which lessens their significance as types of heterocyclic inhibitors (HCIs).

Within the five-membered heterocycles, nitrogen-containing compounds—triazoles, imidazoles, tetrazoles, pyrazoles, and so on—are the significant chemicals that hinder corrosion.^130^ Thiazoles, oxazoles, thiadiazoles, and other heterocycles are examples of compounds that include sulfur or oxygen atoms in addition to nitrogen atoms. Important compounds that suppress corrosion are fused-ring heterocycles such as benzotriazole, indole, benzoxazole, and benzimidazole. Six-membered heterocycles are the most essential type of these inhibitors because they undergo the least amount of ring strain. Tetrazines, pyridine, diazines, triazines, and their counterparts are a few examples of these.^130^ Fused ring quinoline inhibitors are routinely employed found widespread use as corrosion inhibitors.

**Fig. 4:**
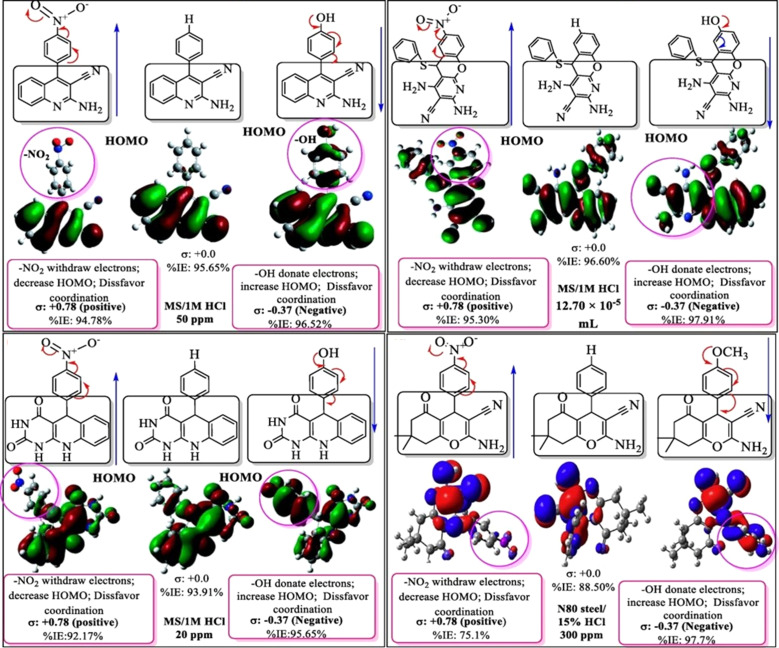
Adsorption and coordination bonding behavior of pyridine, triazine and quinoline and their mono- and di-substituted derivative.^134^

Moreover, the existence of polar groups likes OH, –NH_2_, –OCH_3_, and others, as well as unsaturation through triple and double bonds, allows these inhibitors to readily donate electrons and be protonated in aggressive corrosive environments. Electron-donating substituents such as –OH, –NH_2_, and others, promote the electron density at the active site, facilitating the interaction between the metal substrate and inhibitor molecules.^43,131^ Conversely, electron-withdrawing substituents like –NO_2_, –CN, COOH, and others, minimize the electron density at the inhibitor's active sites, leading to a minimized rate of adherent on metal surfaces.^132,133^ The availability of substituents in the side chain(s) and the number of heteroatoms can determine the formation of poly, mono-, and bi-dentate complexes. The impact of certain common substituents on the inhibition potential of some heterocyclic inhibitors is schematically depicted in Fig. 4. The literature review demonstrated that the values of Δ*G*_ads_ for the adhered of various heterocycle ranges between −40 and −20 kJ mol^−1^, reflecting that their adsorption phenomena predominantly follow the physiochemisorption.

#### 3.2.3. Summary of organic inhibitors

Phosphonic acids are successful corrosion inhibitors because of their commercial availability, low toxicity, and good efficacy. The reason phosphonate inhibitors are employed is because they adsorb on targeted surfaces and create insoluble protective layers that augment activation energy and minimize the active area of targeted metal. These inhibitors have a combination of anti-scaling and anti-corrosion features, as well as a robust protective capability across a variety of salinities and temperatures. However, there are several drawbacks, such as the potential for localized corrosion, minimized performances in areas of stagnant electrolytes, and the existence of hydrogen sulfide, even in minute amounts. Furthermore, phosphonate inhibitors may break down in the presence of oxidizing biocides. Further, heterocyclic agents are also employed as inhibitors in aggressive media. The wide use of inhibitors is attributed to their ease of application, reasonable cost, and efficiency. Studies have demonstrated that inhibitors containing heteroatoms (N, P, O, S) and lone electrons are essential for physisorption or chemical adsorption to form a protective barrier on certain metal surfaces. Inhibitors frequently come into touch with caustic substances including acids, oxidizing agents, and aggressive ions during industrial operations. A heterocyclic structure must be carefully designed to account for various reactions that can make the inhibitor ineffective or exacerbate the effects of corrosion. Functional groups in the structure directly impact its adherence to metal surfaces. Certain functional groups can be added to heterocyclic agents to boost their design and synthesis while minimizing interfering issues. Furthermore, to guarantee safety, it is crucial to do biological toxicity testing before utilizing heterocyclic inhibitors. Table 2 lists some of the common organic corrosion inhibitors along with their important characteristics.

**Table 2:** Characteristics of some representative examples of organic corrosion inhibitors

Inhibitor	Environment	Nature of adsorption	Conc.	Method of corrosion monitoring	IE %	Ref.
Phosphonate anion (PHOS)	Mild steel/SCW	Langmuir/anodic inhibitor	10^−3^ M	WL, SEM-EDX, PDP, EIS, MD	88%	135
Pyridine	Mild steel/1 M HCl	Langmuir/mixed type inhibitor	10^−2^ M	SEM-EDX, PDP, EIS, MD	46%	136
Quinoline			10^−2^ M		64%	
1,10-Phenanthroline			10^−2^ M		80.4	
3-Pyridylaldoxime (3POH)	Mild steel/1 M HCl	Langmuir/mixed type inhibitor	200 ppm	PDP, EIS	94%	137
1-Benzylimidazole	Carbon steel/1 M HCl	Langmuir/mixed type inhibitor	500 ppm	WL, PDP, EIS	82.0%	138
3-Amino-5-mercapto-1,2,3-triazole (AMTA)	Carbon steel/2 M H_2_SO_4_	N. A./mixed type inhibitor	5 × 10^−3^ M	SEM-EDX, PDP, EIS	76.67%	139
Benzotriazole (BZT)	Carbon steel/2 M HCl	Chemisorption/mixed type inhibitor	500 ppm	WL, PDP, EIS, LPR, DFT, CS	78.1%	140
5-Methyl-1*H*-benzotriazole (5MBZT)					91.8%	
3-Amino-5-methylthio-1*H*-1,2,4, triazole (3AMT)					24.8%	
Allyl-6-nitro-1*H*-indazole	Carbon steel (C38)/1 M HCl	Langmuir/mixed type inhibitor	10^−3^ M	WL, PDP, EIS, XPS, SEM	95%	141
1-Ethyl-5-nitro-1*H*-benzimidazole-2-thiol			10^−3^ M		97.5%	
Pyridine	MS/0.1 M HClO_4_	N. A./mixed type inhibitor	0.05 M	PDP, DFT, MD	27.5%	142
2-Amino-5-chloropyridine					68.3%	
2-Amino-3,5-dichloropyridine					52.1%	
2-Amino-3-benzyloxypyridine					42.4%	
Imidazole-4-methylimine thiourea (MIT)	Mild steel/1 M HCl	Langmuir/mixed type inhibitor	200 ppm	SEM, EISPDP, DFT, MD	93.7%	143

### 3.3 Adverse effects of inorganic and organic corrosion inhibitors

Inorganic corrosion inhibitors can have significant adverse effects, particularly on the environment and human health. One of the primary concerns is the environmental impact of these inhibitors. Many inorganic corrosion inhibitors, such as chromates and phosphates, can accumulate in the environment and lead to the contamination of water bodies, soil, and air. Chromates, for instance, are known to be carcinogenic and can pose a serious threat to both ecological systems and human health.^144^ The release of these harmful substances into the environment can have far-reaching consequences, disrupting delicate ecosystems and potentially exposing human populations to toxic substances through various exposure pathways, such as drinking contaminated water, ingesting contaminated foods or inhaling polluted air. In addition to the adverse environmental impact, inorganic corrosion inhibitors can also exhibit direct toxicity to living organisms. Substances like nitrite and zinc salts can be toxic to a wide range of organisms, including aquatic life, plants, and humans, depending on the concentration level and exposure route.^145^ Ingestion or inhalation of these inhibitors can lead to various health problems, such as respiratory issues, skin irritation, and gastrointestinal disorders. This toxicity affects not only the workers who handle these inhibitors but also the general public and the surrounding communities where these substances are used or disposed of.

Further, organic corrosion inhibitors, while offering alternative solutions to inorganic inhibitors, also can have their own set of adverse effects that must be considered. One significant concern is the toxicity and environmental persistence of some organic inhibitors, such as imidazolines and quaternary ammonium compounds. These substances can be toxic to aquatic organisms and may bioaccumulate in the environment, leading to long-term impacts on ecosystems. Another adverse effect of some organic corrosion inhibitors is their potential to cause foaming or fouling in various industrial systems, such as heat exchangers, pipelines, or boilers. This can lead to operational issues, reduced efficiency, and the need for additional maintenance and cleaning, which can be time-consuming and costly. Furthermore, the use or decomposition of organic corrosion inhibitors can result in the formation of potentially harmful byproducts. These byproducts may have adverse environmental or health implications, as they can be carcinogenic, ecotoxic, or have other undesirable properties. The disposal and waste management of spent or unused corrosion inhibitors also present challenges. These substances may require specialized treatment or containment to prevent environmental contamination, and improper disposal can result in the release of harmful substances into the environment, further exacerbating the risks to ecosystems and human health.^146–150^ To address these adverse effects and promote sustainable corrosion management practices, it is crucial to carefully evaluate the specific application, environmental conditions, and potential risks associated with both inorganic and organic corrosion inhibitors before selection and use. Proper handling, disposal, and the development of environmentally friendly alternative inhibitors can help mitigate the associated risks and ensure the safe and effective use of these substances.

### 3.4 Environmentally friendly corrosion inhibitors

#### 3.4.1. Polysaccharides inhibitors

**Fig. 5:**
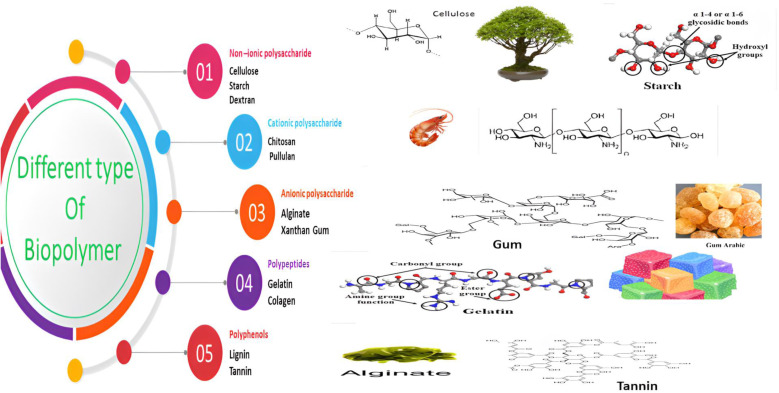
Various types of biopolymers.

**Fig. 6:**
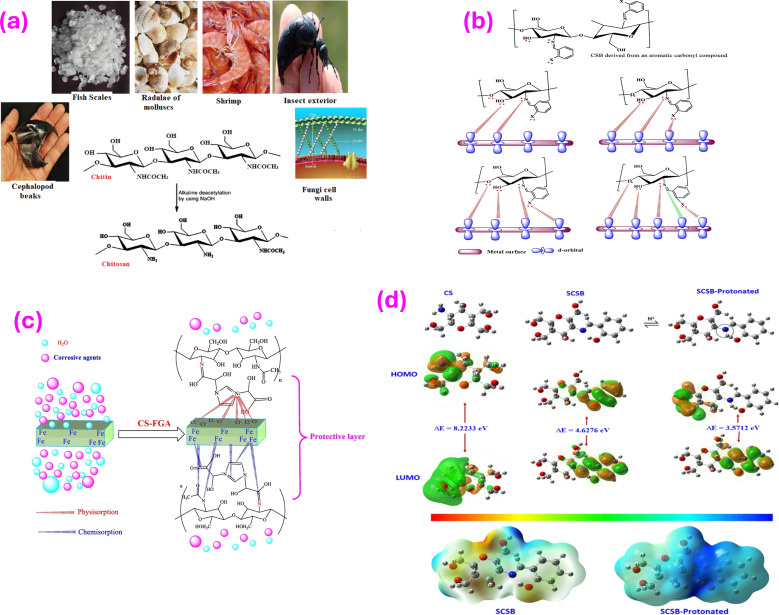
(a) Various sources of chitosan, reprinted with the permission of ref. 156, copyright 2024, Elsevier; (b) illustration of formation of chelating complexes of chitosan Schiff bases, reprinted with the permission of ref. 157, copyright 2024, Elsevier; (c) adsorption mode of CS-FGA molecule on the MS surface, reprinted with the permission of ref. 158, copyright 2024, Elsevier; (d) optimized geometry; HOMO and LUMO orbitals of neutral and pronated SCSB; and MEP images of neutral and pronated SCSB, reprinted with the permission of ref. 159, copyright 2024, Elsevier.

Biopolymers are natural polymers derived from renewable sources like plants, animals, and microorganisms. These materials have garnered attention due to their unique properties and environmental sustainability. Some common examples of biopolymer types include alginate, chitosan, lignin, cellulose, and starch (Fig. 5). These materials possess the ability to form protective layers on metal surfaces, effectively preventing the entry of corrosive chemicals. Biopolymers offer sustainable and effective alternatives to other inhibitors. Their capacity to adhere to metal surfaces and create protective barriers helps to hinder the electrochemical processes that lead to corrosion. The adsorption on surfaces is impacted by various elements, such as pH, polymer concentration, temperature, and surface charge. Through hydrogen bonding, electrostatic forces, and other molecular interactions, biopolymers can effectively block the penetration of corrosive substances. The utilization of carbohydrates as inhibitors presents an attractive alternative due to their abundance, renewability, and low environmental impact. Carbohydrates can be derived from numerous sources, including plant materials (*e.g.*, starch and cellulose), marine crustacean shells (*e.g.*, chitosan and alginate), and even from bacterial fermentation processes.^151–153^ To optimize the corrosion inhibition properties of carbohydrates, chemical modifications can be employed. By targeting specific functional groups, such as hydroxyl, carboxyl, or amino groups, within the carbohydrate structure, their inhibitory effects can be enhanced. For instance, the introduction of amino groups can improve adsorption characteristics, facilitating the formation of more effective protective films and increasing corrosion resistance. Numerous studies have investigated the corrosion inhibition potential of different carbohydrates for several metals, including steel, in various corrosive environments such as acidic, alkaline, and saline solutions.^154,155^ Chitosan (CS), a derivative of chitin found in crustacean shells, has also gained attention as an effective corrosion inhibitor. Chitin is derived primarily from shrimp and other crustacean shells (Fig. 6a). For the partial deacetylation of Chitin, it is common to employ an alkaline solution. With their remarkable qualities, both chitin and CS prove to be exceptional candidates for a wide array of biological and industrial applications, including the prevention of corrosion. Chitosan consists of various reactive groups: a primary CH_2_OH, a secondary CH–CH_2_OH, and an amino group (–NH_2_), attached to carbon atoms.^153^ The use of CS as a corrosion inhibitor is justified for various reasons; for instance, owing to its polymer nature, power to fully adsorb onto and cover various metallic surfaces, and efficient corrosion protection.^157,160^ In fact, these biomacromolecules contain polar moieties that can act as sites for adsorption when they interact with metal surfaces. Chitosan, a versatile material, exhibits both physisorption and chemisorption properties. Chemisorption occurs through the sharing of electrons between the N and O atoms of CS, leading to coordination bonding.^161,162^ Physisorption, on the other hand, is driven by electrostatic interactions between the charged CS and surface.^163^ Polar groups like –OH and –NH_2_ have the capacity to be protonated in aqueous solutions, which may contribute to the creation of cationic forms.^164^ On the other hand, the buildup of counter ions from the electrolyte ions at the positive surface causes the metal surface to turn negatively charged. The interplay between charge interactions and chemisorption and physisorption adds to chitosan's adaptability and adsorption power across a range of applications.^158^ The natural antimicrobial features of CS can help prevent MIC by limiting the growth of corrosive microorganisms on metal surfaces. Its diverse inhibitive mechanisms contribute to its performance in various corrosive environments.^134^ Several factors including concentration level, pH, duration of exposure, and temperature may impact the performance of CS.^134^ For instance, when applied to MS protection, CS exhibited 96% and 93% corrosion inhibition efficiency at 60 °C and 70 °C, in an HCl environment.^165^ Using polarization analyses, it was proposed that CS served as a mixed inhibitor, affecting both the cathodic and anodic reactions. Impedance measurements reflected that CS adsorption occurred at the solution/metal interface. The Langmuir isotherm model was noticed to agree with CS adherence onto MS surfaces. Thus, when 200 ppm CS was employed as a corrosion inhibitor in a 1 M sulfamic acid medium, there was significant inhibitory performance of 73.8% noticed for MS.^166^ The research demonstrated that CS operated by obstructing the active sites of the metal, thus mimicking both the anodic and cathodic processes that take place on the surface of tested MS. To further confirm this, surface examinations using atomic force microscopy (AFM) and SEM were conducted, revealing the creation of a protective film on steel surface owing to the existence of the inhibitor. Additionally, the study determined that the CS adsorption adhered to the Langmuir model. However, a major drawback of CS is its inadequate solubility in many liquids, which greatly limits its use as a corrosion inhibitor.^154^ CS has a unique property – it is insoluble water, but it can dissolve in a slightly acidic medium (with a pH below 6.5). This is owing to –NH_2_ in the CS converting to a soluble glucosamine-NH_3_^+^ form under these mildly acidic conditions.^134^ The solubility of CS in aqueous electrolytes is robustly impacted by the level of deacetylation (% DD) – the robust the % DD, the greater the solubility.^134,167^ Commercially useful grades of CS typically have a % DD between 60–100% and a molecular weight range of 3800–20000 Da.^168,169^ Researchers have been actively exploring approaches to further enhance the solubility of CS. One common approach is to chemically modify the CS structure by introducing more polar substituent groups, such as ester (–COOR), hydroxyl (–OH), amino (–NH_2_), ether (–O–), nitrile (–CN), amide (–CONH_2_), and nitro (–NO_2_) groups.^48,49^ These phenomena can make soluble better by improving the bond between the changed CS and the water, or by turning the CS into even more watery forms.^161,170,171^ For instance, To enhance chitosan's solubility, a chitosan salt was fabricated and tested as an effective corrosion inhibitor for N80 steel in seawater.^161^ Results revealed that CS served as a mixed-type inhibitor with a notable inhibitory impact on the anodic reaction. Notably, at a concentration of 1000 ppm, CS displayed an impressive inhibition performance of 96.68%. Currently, there is a significant focus on the chemical bonding of organic compounds with CS and their potential as agents to mitigate corrosion. To achieve this, CS undergoes an operation called cross-linking, wherein an organic polyol-linker is employed to connect multiple CS chains. This type of cross-linking boosted the solubility and protective capabilities of the CS derivatives. Therefore, the use of polyethylene glycol (PEG) cross-linked-CS was tested as a corrosion inhibitor for steel, at a dosage of 200 mg L^−1^, and exhibited a high inhibition performance of 93.9%.^172^ The adherence of CS-PEG on the steel surface and the generation of a protective film were accountable for the significant enhancement in surface smoothness noticed at the optimal CS-PEG level. The transfer of d-orbital electrons from iron to unoccupied antibonding orbitals of CS inhibitor promoted adherent phenomena, while heteroatoms with free lone pair electrons present on employed CS inhibitor supported chemical adherence.

Various works reported that chitosan-based Schiff bases (CSBs) exhibit outstanding stability and resist deterioration. The fact that CSBs are composed of polymers, which enables them to offer superior covering and protection for metal surfaces, is one of its benefits. Additionally, polar substituents found in CSBs can serve as adsorption sites when they engage and form bonds with metal surfaces. Furthermore, these substituents aid in making CSBs more soluble in electrolytes containing water. It's vital to remember that CS may be made more soluble and corrosion-resistant by altering its structure. As seen in Fig. 6b, CSBs are able to make powerful connections with metal surfaces because they have several electron-rich moieties.^157^ Moreover, in this bonding process, the imine bond (>C=N–) is very significant. For instance, Chitosan-Salicylaldehyde-Schiff base (CS-SB) was investigated as a corrosion inhibitor of steel in 3.5% NaCl solution saturated with CO_2_.^159^ The researchers found that the existence of CS-SB significantly enhanced the surface morphologies of the steel, as noticed through SEM and AFM analysis. Additionally, the results from PDP tests reflected that CS-SB served as a mixed-type inhibitor, effectively minimizing the corrosion process. At a level of 150 mg L^−1^, CS-SB exhibited an inhibition performance of 95.2% and a corrosion rate of 0.444 mm per year. Among the isotherm models tested, the Langmuir model offered a great fit for the experimental data. Luo and colleagues^158^ developed a CS-FGA through a straightforward amidation reaction. The protonated CS-FGA molecules with –N^+^= species can physically adhere to the pre-adsorbed Cl^−^ ions. Additionally, the heteroatoms and polar groups in the CS-FGA can accept and donate electrons, leading to chemical adsorption through electron mobility between the Fe atoms and heteroatoms or the polar groups. The adsorption mode of CS-FGA is illustrated in Fig. 6c. Furthermore, SCSB was fabricated, and its inhibition impact was analyzed using various approaches. Computational investigation revealed the excellent adsorption of the protonated form of the inhibitor (Fig. 6d). The inclusion of the salicylaldehyde moiety has significantly promoted the size of the resulting SCSB molecule, leading to a planar structure. The enhanced structural planarity and size are likely to promote the coverage of the surface.^159^

On the other hand, cellulose is a valuable carbohydrate-based polymer with various applications. It consists of β-d-glucose units linked by a β-(1–4)-glycosidic bond to form a linear chain polysaccharide. Cellulose is a key component of Oomycetes, plant cell walls, and algae. It is abundantly found in nature, with approximately 40–50% found in wood, 90% found in cotton, and 57% in hemp.^173^ Carboxymethyl cellulose (CMC) is one of the derivatives of cellulose that contains carboxymethyl bonded to the glucopyranose units in the structure.^174^ For instance, cellulose extracted from pistachio nut shells was employed as a corrosion inhibitor of steel.^175^ The researchers found that the inhibitor adhered to the Langmuir isotherm and exhibited a mixed-type inhibition behavior. Furthermore, 800 ppm level of inhibitor resulted in a significant 92% performance. Additionally, the inhibitor exhibited both chemisorption and physisorption characteristics onto the tested metal surface. Furthermore, the use of hydroxyethyl cellulose (HEC) as a corrosion inhibitor for A1020 steel in an acidic medium was investigated.^176^ The researchers found that the inhibition performance was promoted with higher HEC levels but minimized with rising temperature. HEC was identified as a mixed-type inhibitor, mainly anodic, based on PDP investigations. In an acidic medium, HEC exists in a protonated form and interacts electrostatically with Cl^−^ ions already adherent to the metal surface. Protonated HEC competes with H^+^ ions for electrons on the surface, resulting in the release of H_2_ gas. This phenomenon returns HEC to their neutral form and promotes chemisorption by oxygen atoms, which have a free lone pair of electrons. Additionally, the accumulation of electrons on the steel surface leads to a more negative charge. As a result, an electron from the d-orbital of Fe may move to a vacant antibonding orbital of the inhibitor. Similar, carboxymethyl cellulose (CMC) was employed as an inhibitor for steel in a 3.5% NaCl medium saturated with CO_2_.^177^ The inhibitor's adherence obeyed Langmuir's isotherm. In another study, the researchers examined how the addition of halide (Cl^−^, Br^−^, and I^−^) impacted the performance of CMC in an acidic medium.^178^ They noticed that the existence of iodide ions promoted the inhibition provided by CMC, resulting in a synergistic impact. However, the existence of chloride had the opposite impact, causing an antagonistic impact on inhibition. The effectiveness of CMC, as displayed by the inhibition efficiency (% *η*) value, was promoted over time as the MS was immersed in the medium containing CMC and iodide ions, as well as other halide ions.

On the other hand, lignin is a 3D aromatic biopolymer that can be found in a variety of materials, including bagasse, wood, and sugar cane. It is ideally suited as coating and corrosion inhibitor owing to its accessibility, environmental friendliness, and anti-corrosion features.^179–181^ While lignin already possesses natural functional groups (*e.g.*, aromatic sites, –OH, and –OCH_3_) that contribute to its beneficial properties, efforts have been made to introduce new functionalities to enhance its anticorrosion potential.^182,183^ These polar functional groups within lignin are particularly essential for its corrosion suppression mechanism, as they facilitate adherence onto metallic surfaces.^184,185^ Despite lignin's demonstrated corrosion inhibition properties, ongoing research focus on exploring various strategies to further enhance its effectiveness as a corrosion inhibitor.^186–188^ For instance, lignin as an inhibitor was applied to ferrous metal surfaces exposed to acidic conditions.^189^ Lignin is obtained from bagasse through an acidification approach and its functional groups are confirmed through FTIR analysis. The researchers evaluate the corrosion inhibition performance of lignin by varying its levels and the duration of immersion. Through the weight-loss approach, they demonstrated that the most optimal inhibition performance was achieved with a lignin level of 10 g L^−1^ and a metal immersion time of 6 hours, resulting in an impressive 80.79% corrosion inhibition. The Langmuir adsorption isotherm described the adherence of lignin onto the surface of tested MS.^190^ Further, the research showed that the existence of lignin can significantly impact the texture and features of the target metal surface. SEM images have revealed that when lignin was added to a solution, it effectively minimizes the roughness of the metal surface, leading to enhanced homogeneity and smoothness. This is a strong indication of lignin's potential in inhibiting corrosion consequently minimizing the oxidative metal dissolution.^191,192^ Furthermore, AFM analysis has demonstrated that in the absence of lignin inhibitors, the surface undergoes deterioration due to acidic conditions. However, the addition of lignin inhibitors to the solution successfully mitigates surface corrosion. Lignin has been found to diminish the maximum height scales of surface roughness when compared to samples without inhibitors.^180,193^Table 3 lists some of the common polysaccharide corrosion inhibitors along with their important characteristics.

**Table 3:** Extract of data of some polysaccharides evaluated as corrosion inhibitors

Inhibitor	Environment	Nature of adsorption	Conc.	Method of corrosion monitoring	IE %	Ref.
Chitosan	Mild steel, 0.1 M HCl	Langmuir	1.8 mM	WL, EIS, PDP, AFM and EDS	92.1	194
Chitosan	1 M HCl, carbon steel	Langmuir	5000 ppm	WL	93.2	195
CH + KI	Mild steel, 1 M sulfamic acid	Langmuir	200 ppm CH + 5 ppm KI	WL, EIS, PDP, AFM and SEM	91.6	166
Chitosan + KI	15% H_2_SO_4_	Langmuir	5 g CH + 5 mM KI	WL, DEIS, PDP, AFM, SEM and EDS	97.6	196
Chitosan–polyaniline (PANI/CTS)	0.5 M HCl, mild steel	Mixed-type inhibitor	200 ppm	EIS, PDP, SEM and DFT	79.02%	197
Chitosan Schiff base (ChTSB)	Mild steel, 1 M HCl	Temkin isotherm, mixed-type	1500 ppm	EIS, PDP, AFM, SEM and EDS	86.94%	198
Salicylaldehyde-chitosan Schiff base (SCBS)	Carbon steel (J55 steel), 3.5% NaCl	Langmuir, mixed type	150 ppm	XPS, EIS, PDP, AFM, SEM and EDS	95.4%	159
CS-2	Mild steel & 1 M HCl	Langmuir, mixed type	150 ppm	XPS, EIS, PDP, MD, AFM, and SEM	98.0%	154
Chitosan-cinnamaldehyde Schiff base	Carbon steel & 3.5% NaCl	Langmuir, mixed type, cathodic predominantly	600 ppm + 10 mM KI	DFT, EIS, PDP, MD, SEM and EDS	92.67%	199
4-(Dimethylamino)benz-aldehyde-chitosan	Mild steel/1 M HCl	Langmuir	50 ppm	PDP, EIS, WL, SEM and EDS	90.65	200
Carboxymethyl chitosan (CMC)	3.5% NaCl, carbon steel	Langmuir, mixed type inhibitor	80 ppm	PDP, EIS	85.57% 5	201
Chitosan (CH) carboxymethylcellulose (CMC)	5% NaCl + CO_2_aPI 5 L X60 pipeline steel	Langmuir, mixed type inhibitors	100 ppm	EIS, PDP and SEM	45%	177
			100 ppm		39%	
Ethyl hydroxyethyl cellulose (EHEC)	1 M H_2_SO_4_/mild steel	Langmuir, slightly cathodic	2500	WL, EIS, PDP and DFT	68.19%	202
Carboxymethyl cellulose (CMC)	2MH_2_SO_4_/mild steel	Langmuir	500 ppm	WL and hydrogen evolution	65%	178
Exudate gum from *Araucaria heterophylla* tree (AH-gum)	Mild steel/1 M H_2_SO_4_	Langmuir, mixed type	0.05% v/v	WL, EIS, PDP, DFT, MD, AFM, SEM	79%	203
Exudate gum from Terminalia mentaly tree (TM-gum)	Carbon steel/1 M HCl	Langmuir and Temkin, mixed type	2000 ppm	WL, EIS, PDP, SEM	96%	204
Mangifera indica gums tree (MA-gum)	Carbon steel/1 M HCl	Langmuir and Temkin, mixed type	1000 ppm	WL, LP, OP	98%	205
Gellan gum	Cast iron/1 M HCl	Langmuir, mixed type	5000 ppm	WL, PDP, EIS, SEM	81%	206
Gum acacia	Mild steel/1 M HCl	Langmuir, mixed type	1000 ppm	WL, PDP, EIS	97%	207
Xanthan gum (XG)	Mild steel/1 M HCl	Langmuir, mixed type	1000 ppm	WL, PDP, EIS, SEM	74.24	208

Alginate, a naturally occurring polysaccharide derived from brown seaweed, has emerged as a promising and eco-friendly inhibitor.^209^ As industries and researchers seek alternatives to traditional and hazardous corrosion inhibitors, the use of alginate has gained significant attention due to its unique properties and eco-friendly nature.^210^ This biopolymer derived from brown seaweed, with its unique structure imparts sodium alginate (SA) many desirable features, such as biodegradability, robust solubility, non-toxicity, and biocompatibility. Recent works have highlighted the successful employing of SA as a novel and powerful biological inhibitor for MS in challenging environments, including saline media and hydrochloric acid pickling.^211,212^ The primary mechanism by which alginate inhibits corrosion is the formation of a protective, passivation layer on the metal surface. Alginate's long, linear polysaccharide chains are composed of two main types of monomer units: guluronic acid (G) and mannuronic acid (M).^213^ These monomers possess various functional groups, such as carboxyl and hydroxyl groups, which allow alginate to adsorb strongly onto metal surfaces through chemical and physical interactions.^210,214^ The adsorption of alginate on the metal surface creates a barrier that separates the metal from the corrosive environment, effectively shielding it from direct contact with corrosive agents like oxygen, moisture, and attacking ions.^215^ This physical barrier inhibits the electrochemical processes that drive corrosion, slowing down the anodic and cathodic reactions responsible for metal dissolution and the formation of corrosion products.^216^ Namely, Alginate's corrosion inhibition mechanism involves the modulation of both the anodic and cathodic reactions that occur during the corrosion process. The adsorption of alginate on the metal surface can block the active sites and alter the kinetics of both of the anodic (metal dissolution), and cathodic (oxygen reduction) reactions, thereby reducing the overall corrosion rate.^211,217^ Moreover, the adsorption of alginate on the metal surface can promote the formation of a stable, protective passive film. This passive film acts as an additional barrier, further enhancing the corrosion resistance of the metal. The synergistic effect of these multifaceted mechanisms, including adsorption, chelation, anodic and cathodic inhibition, and pH buffering, contributes to the exceptional corrosion inhibition performance of alginate. The understanding of these underlying mechanisms is crucial for the rational design and optimization of alginate-based corrosion inhibitors, enabling their effective deployment in a wide range of industrial applications. For instance, using polarization studies, the SA mitigation performance on MS in 1 M HCl medium was explored and revealed an impressive performance of 90.9% inhibition at a level of 1500 mg L^−1^ SA, highlighting its potential as a powerful inhibitor in acidic medium.^218^

Furthermore, natural gums are complex polymers composed of long polymeric sugar chains with enormous molecular weights, which are formed of monosaccharide units joined by glycosidic linkages.^219,220^ Based on their environmental friendliness and wide availability, natural gums have drawn attention in corrosion inhibitor research. These natural gums' molecular weight, chemical makeup, and molecular and electrical structures all have a significant impact on how well they prevent corrosion.^221^ These gums have chemical components that prevent corrosion by creating hydrophobic barriers, which limit the entrance of molecules and ions that cause corrosion at the metal–solution interface. Furthermore, certain gums have polar functional groups that attach to metal surfaces to promote protection by improving electron or charge transport.^221,222^ The performance of natural gums as inhibitors is significantly influenced by temperature and duration of immersion.^223^ However, natural gums do not show remarkable inhibition when employed in their pure form because of things like quick hydration, microbial and algal contamination, pH-dependent solubility, and heat instability.^224^ A variety of modification techniques—which fall into the categories of physical or chemical strategies—are used to get over these obstacles.^225^ To provide a synergistic impact, physical tactics entail combining and mixing natural gums with materials like surfactants and halides. Conversely, the chemical approach makes use of natural gums' many qualities to enhance their inhibitory qualities.^225^ This method frequently makes use of procedures including grafting, crosslinking, esterification, and etherification. Creating composites and nano-composites—wherein inorganic components at the nanoscale are integrated into the gum matrix—is a more modern technique for modification.

For instance, several gum types were studied as mild steel corrosion inhibitors, including AF-gum (*Albizia ferruginea* gum), KS-gum (*Khaya senegalensis* gum), Daniella Oliverri gum, AL-gum (*Anogessus leocarpus* gum), FTP-gum (*Ficus tricopoda* gum), CS-gum (*Canarium schweinfurthii* gum), KI-gum (*Khaya ivorensis* gum), FP-gum (*Ficus platyphylla* gum), and CA-gum (*Commiphora Africana* gum).^225–230^ In general, as the dosage is increased, their inhibitory performances get better, but when the temperature of the solution increases, they get worse. The inhibitory property arises from the adsorption of heteroatom-rich molecules, which act as the anchor point for the adsorption process.^225^ The inhibition efficiency falls between 66% and 84%. They adsorb spontaneously, according to the physical adsorption mechanism, and their ideal concentration is 0.50 g L^−1^.

#### 3.4.2. Amino acid and proteins inhibitor

Proteins have gained attention as inhibitors owing to their low environmental impact. They offer features such as high effectiveness, low toxicity, and biocompatibility. Proteins can generate a protective layer on the treated metal surface, hindering corrosion processes.^231^ Apart from their crucial functions such as catalyzing metabolic reactions and DNA replication within living organisms, various proteins have been explored for their inhibitory features in various corrosive environments.^232^ On the other hand, amino acids, the building units of proteins, are organic compounds that are characterized by specific side chains unique to each amino acid, where these side chains consist of functional groups such as carboxyl groups (–COOH) and amino groups (–NH_2_), among other groups. The structure of amino acids includes elements such as nitrogen, oxygen, hydrogen, carbon, and possibly other atoms that are present in the side chains.^233^ Peptide bonds, also known as amide bonds, form when the –COOH of one amino acid molecule reacts with the –NH_2_ of another amino acid molecule.^234^ According to studies, side chains containing “R” groups and a large number of linked amino acids form polypeptides.^235^ Ultimately, polypeptides come together to form linear biopolymers known as proteins.

**Fig. 7:**
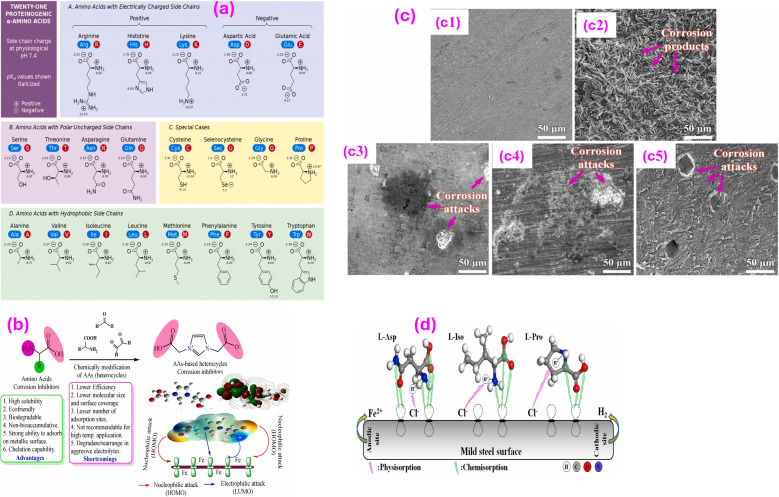
(a) Chemical structure and classification of amino acids, reprinted with the permission of ref. 241, copyright 2024, Elsevier, (b) advantages and limitations of AA-based heterocyclic and bare amino acids, reprinted with the permission of ref. 241, copyright 2024, Elsevier; (c) surface morphology of Ms: (c1) before immersion, and after immersion in: (c2) 0.5 M HCl, (c3) l-Asp, (c4) l-Iso, and (c5) l-P, reprinted with the permission of ref. 242, copyright 2024; (d) inhibition mechanism of l-Asp, l-Iso, and l-Pro inhibitors in 0.5 M HCl solution, reprinted with the permission of ref. 242, copyright 2024.

The impacts of casein on the corrosion behavior of steel in an acidic medium were investigated using various analytical techniques.^236^ The WL results reflected that as the level of casein increased from 50 to 400 ppm, the rate of corrosion inhibition declined from 34.3 mpy to 3.9 mpy. However, when the temperature of the test medium was raised from 298 K to 343 K, the surface coverage and consequently the casein inhibition role were negatively impacted. Thermodynamic calculations displayed that in the presence of 400 ppm casein, the enthalpy of activation and activation energy enhanced from 5.95 kJ mol^−1^ to 43.23 kJ mol^−1^ and from 8.61 kJ mol^−1^ to 45.89 kJ mol^−1^, respectively. The functional groups of casein enhanced electrostatic adherence to the employed metal surface (physisorption). Additionally, in the chemisorption operation, heteroatoms in casein, such as oxygen, donated their lone-pair electrons to the metal-vacant d-orbitals. In another investigation, EIS was used to explore the behavior of steel in an HCl solution in the presence of gelatin.^237^ It was noticed that the existence of gelatin led to the creation of a protective layer on the surface of the tested MS, which slowed down the charge movement reaction between the medium and metal. Gelatin was found to serve as a mixed-type inhibitor, blocking both the cathodic and anodic sites, and its adsorption features followed the Langmuir model. The polypeptide chain's backbone amide linkages were believed to account for gelatin adherence onto the metal surface. It is noteworthy to emphasize that the number of papers on the anticorrosive behavior of proteins is comparatively lower than that on the performance of amino acids (AAs) in inhibiting corrosion.^238^ The AAs can be viewed as comparatively more affordable, soluble, and environmentally friendly substitutes.^239,240^ Besides the environmental aspects, the inclusion of heteroatoms such as sulfur (S), nitrogen, conjugated π-electron systems, and oxygen (O), plays an essential role in the performance of amino acid inhibitors. Amino acids can be classified into three main groups: acidic, neutral, and basic (Fig. 7a).^241^ The classification is based on the relative abundance of amino (–NH_2_) groups and carboxyl (–COOH) within the amino acid structure. Basic amino acids contain more –NH_2_ groups than –COOH groups, while acidic amino acids exhibit the opposite trend.^241,243^ Neutral amino acids are characterized by a balanced ratio of amino to carboxyl groups. This unique structural feature of amino acids suggests their potential as powerful inhibitors. The adsorption mechanism of these substances on MS is particularly intriguing, granting them the remarkable ability to function as natural inhibitors. The adsorption phenomenon is driven by the binding of the N atom within AAs to the MS. This adsorption phenomenon facilitates the creation of a robust layer on the metal surface, effectively slowing down the rate of corrosion.^241,242^ The physisorption interaction will occur more quickly and effectively with amino acids than with traditional inhibitors because they are already charged particles. Furthermore, it is possible to propose that amino acids have at least two adsorption sites, while traditional inhibitors only have one by treating charged moieties as adsorption centers. Additionally, some specialized subgroups of amino acids, called amino alcohols, have been found to have remarkable corrosion inhibition performance.^238,241,244^ They achieve this through a two-pronged strategy. First, they replace chloride ions that play a critical role in the corrosion phenomenon. Second, they block specific locations where O_2_ gains electrons, thus hindering overall corrosion. This dual approach allows these AAs to be promising inhibitors.

For instance, the ability of leucine, alanine, and glycine to suppress steel corrosion in an acidic medium was analyzed, where Langmuir's isotherm best fitted amino acids adsorption on steel.^245^ Furthermore, AAs and their derivatives such as heterocyclic amino acids substances can also form powerful chelating complexes that excellently bind to metal surfaces.^246^Fig. 7b shows the advantages and limitations of AA-based heterocycles and bare amino acids as corrosion inhibitors.^241^ Specific amino acids like tryptophan, histidine, and proline, as well as their derivatives, have been widely utilized for corrosion hindering owing to their capacity to bind to surfaces and create these chelating complexes.^247^ For instance, proline can form a bidentate chelating complex employing its carboxyl and amino groups.^248^ Tryptophan and histidine can form even robust tridentate chelating complexes owing to another N in their indole and imidazole structures, respectively.^249^ The adsorption phenomenon of these compounds primarily follows the Temkin and Langmuir adsorption isotherms, and they function as mixed-type inhibitors. While amino acids substances are known for their ability to coordinate with substrates, they are generally not recommended for commercial uses owing to their relatively lower molecular size and small coverage of surface. However, the heterocyclic substances derived from amino acids often have acceptable molecular sizes, allowing them to more effectively protect surfaces.^241^ The semi-synthetic nature of the heterocyclic substances derived from AAs is believed to make them more friendly alternatives to conventional, harmful inhibitors. For example, a separate study demonstrated the ability of the amino acid proline to minimize corrosion. Consistent with expectations, the researchers noticed that rising the pH from 2 to 5 led to an improvement in the inhibition performance, rising from 16% to 65%.^248^ The corrosion inhibition of l-isoleucine (l-Iso), l-proline (l-Pro) and l-asparagine (l-Asp) on steel immersed in 0.5 M HCl was also investigated.^242^ At 1000 ppm, l-Pro, l-Iso and l-Asp showed 91%, 93% and 95% inhibition, respectively. Fig. 7c shows the surface morphology of MS before and after immersion of each inhibitor at 1000 ppm. XPS studies showed that the acceptor–donor interactions between surface iron atoms and AAs heteroatoms play a key role in the chemisorption phenomenon (Fig. 7d).^242^ Furthermore, the inhibition capabilities of unmodified proline against corrosion have been explored.^250,251^ Likewise, the bare amino acid histidine is commonly utilized, solely or alongside other substances designated as synergists, as a method of combating corrosion.^252–254^ Tryptophan (Trp) possesses a unique chemical composition, featuring an indole group integrated within its side chain. This indole group is formed by the fusion of a benzene ring and a pyrrole ring. Due to this distinctive structural attribute, Trp has the potential to serve as a more robust inhibitor compared to histidine (His) and proline (Pro). Consequently, numerous studies have been dedicated to exploring the role of Trp in mitigating corrosion in aqueous environments.^255,256^Table 4 lists some of the common amino acids-based corrosion inhibitors along with their important characteristics.

**Table 4:** Extract of data of some amino acids and proteins evaluated as corrosion inhibitors

Inhibitor	Environment	Nature of adsorption	Conc.	Method of corrosion monitoring	IE %	Ref.
Glycine	Mild steel/0.5 M HCl	N. A.	N. A.	PDP	53.9%	257
Phenylalanine					74.8%	
Glutamic acid					8.2%	
Glutamic acid with Zn^2+^	Carbon steel/sea water	N. A.	200 ppm glutamic acid with 25 ppm Zn^2+^	WL, EIS, PDP, AFM, SEM	87%	258
Methionine methyl ester (MME) methionine ethyl ester (MEE)	Iron/9 g L^−1^ NaCl	Frumkin model	10^−2^ M	PDP	80%	259
					40%	
					20%	
Poly(vinyl alcohol-histidine)	Mild steel/1 M HCl	Temkin isotherm/mixed-type inhibitor	0.6% wt	WL, EIS, PDP, SEM	95%	260
Cysteine	Mild steel/1 M HCl	Langmuir	0.1 M	MD, EIS	90.4%	261
Polyaspartic acid	Mild steel/0.5 M H_2_SO_4_	N. A.	2000 ppm	WL. PP. EIS. SEM. XPS. FT-IR.	88%	262
Glutamine	Mild steel/1 M HCl	Langmuir	100 ppm	PDP, SEM	96%	263
OPEM	Mild steel/15% M HCl	Langmuir/mixed-type inhibitor	200 ppm	PDP, SEM, EIS	97.5%	264
OPEA					95.5%	
Phenylalanine (PA) + Zn^2+^ ions	Carbon steel/Well water	N. A., anodic inhibitor	5 ppm Zn^2+^ and PA 150 ppm	WL. PDP. EIS. SEM-EDX.	90%	265
Tryptophan (Try), tyrosine (Tyr), serine (ser)	Low alloy steel/0.2 M ammoniated citric acid	Temkin isotherm	0.06 M	PDP. EIS. EFM. OM.	86%	256
					83%	
					82%	
Tetra-*n*-butyl ammonium methioninate	Mild steel/1 M HCl	Frundlich/mixed-type inhibitor	1.59 × 10^−3^ M	OCP. PP. SEM-EDX.	95.1%	266
Casein	Mild steel/0.1 M HCl	Langmuir	400 ppm	WL. PDP. EIS. AFM, FTIR SEM-EDX.	96.41	236
Casein	Steel/0.1 mol L^−1^ NaOH		9.7 × 10^−4^ M	PDP. EIS	97.76%	267
Gluten hydrolysate	Mild steel/1 M HCl	Physical and chemical adsorption/mixed-type inhibitor	1000 ppm	WL. PDP. EIS, FTIR	96.6%	268
Maize gluten meal extract	Carbon steel/1 M HCl	Langmuir	2000 ppm	PDP. EIS, SEM-EDS	88%	269

#### 3.4.3. Polyphenols inhibitors

Tannins are complex combinations of organic polyphenolic substances isolated from plants.^270,271^ They can be classified into two main types: hydrolyzable and condensed tannins. Hydrolyzable tannins originate mostly from pods and fruits, while condensed tannins are found in significant amounts in the wood and bark of various trees such as black wattle.^271,272^ These condensed tannins have been extensively studied as corrosion inhibitors in different environments.^273,274^ The researchers attributed the tannins' inhibitory effect to their electron-rich heteroatoms and double bonds, which facilitate adsorption on the metal surface, thereby contributing to the inhibition process.

## 4. Synergistic inhibition effects

Herein, the term synergy refers to the collaborative impact of multiple corrosion inhibitors working together to promote their ability to hinder corrosion. When these inhibitors are combined, their combined impact is more robust than when employed individually. This collaborative impact can be attributed to the many ways in which these inhibitors work, which complement and promote each other's actions. By employing these inhibitors together, they offer extra protection to the targeted metal surface by combining and/or boosting their mechanisms. Higher doses may be needed when using an individual inhibitor to reach the necessary level of inhibition. Nevertheless, it can be employed in synergy with another inhibitor to accomplish the necessary inhibiting impact at fewer dosages, saving costs in various applications. Further, the development of novel molecules or complexes amongst the inhibitors may result in synergistic interactions. These complexes can be formed either by the inhibitors interacting with the metal surface or by their chemical interactions with each other. Furthermore, synergism is a useful strategy for boosting inhibitors' suppressive potency and expanding their use in harsh circumstances. It is vital for both real-world use and the theoretical investigation of corrosion inhibitors. As mentioned in eqn (1), a synergism parameter (S1) is frequently employed to assess this impact:^275^1
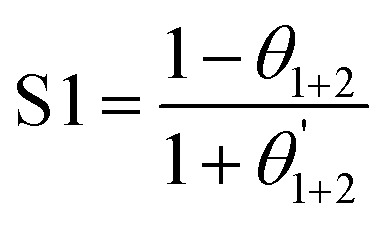
The *θ*_1+2_ parameter is estimated by = (*θ*_1_ + *θ*_2_) − (*θ*_1_ × *θ*_2_), where *θ*_1_ represents the surface coverage of one employed inhibitor, while *θ*_2_ is coverage of another inhibitor or other additive, and 
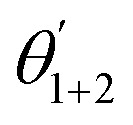
 represents the sum of both employed materials. Whereas a value of S1 less than unity implies an antagonistic impact, a value larger than unity implies inhibitory synergism between the employed materials.

### 4.1 Synergistic impact between inorganic substances

Numerous research studies have consistently shown that achieving the desired level of corrosion inhibition often requires the use of high concentrations of inorganic substances, leading to significant costs. Additionally, the toxicity of some inorganic compounds raised environmental concerns. However, extensive investigation has been conducted in various scientific papers to explore the benefits of utilizing a combination of two or more compounds, Table 5. This synergistic effect has been demonstrated to enhance inhibition efficiency, enabling the usage of lower concentrations of inhibitors compared to employing a single compound even in harsh circumstances. For instance, the combined action of nitrite with phosphate, chromate, zinc, or other inorganic inhibitors has been extensively examined, demonstrating significant synergistic effects. These investigations have found that mixed inhibitors containing nitrite are highly effective, even in the presence of elevated chloride levels.^281,282^ The effectiveness matches that of using nitrite as a standalone inhibitor at higher concentrations. Further, growing environmental consciousness regarding the hazardous impact of nitrite ions has led to the need to reduce their usage. To address this concern, the blending of nitrite with non-toxic and eco-friendly inhibitors like molybdate is being pursued to minimize harmful impacts.^58^ In one study, the impacts of zinc, nitrite, and molybdate ions on the protection of MS in chloride-containing water were examined.^283^ The results demonstrated that molybdate effectively hindered corrosion with pH levels above 6. Similarly, nitrite prevents corrosion at pH levels of 4.5 and above but enhances corrosion below pH 4.5, regardless of the presence of cupric ions. However, when nitrite and molybdate are combined, they served synergistically as inhibitors with or without cupric ions at pH levels of 4.5 and above. This combination also minimized corrosion phenomena in the acidic range between pH 3.0 and 4.5, with a low level of molybdate and high levels of nitrite. The synergistic impact is accomplished by absorbing molybdate which protects the surface passive layer from aggressive anion attacks. To analyze the passive films created on MS during immersion in NaCl medium and high levels of either MoO_4_^2−^ or NO_2_^−^, researchers employed an XPS analysis.^284^ The results confirmed that both anions led to the development of nanometer-thick films (approximately 5 nm) on the surface, with Fe^3+^ ions being the dominant cation. The analysis suggested that an upper sub-layer consisting of Fe_2_(MoO_4_)_3_ was formed, followed by a layer of ferric hydroxide/oxide, may be γ-Fe_2_O_3_. XPS data from the film created by NO_2_^−^ inhibitor supported the notion that it mainly comprised materials like γ-Fe_2_O_3_. Thus, the impact of NaNO_2_ and Na_2_MoO_4_ concentrations on inhibition performance was examined.^58^ At a level of 2 g L^−1^, they achieved 65% and 53% inhibitions, respectively, when these inhibitors were employed individually. Interestingly, upon combined use, a synergistic impact was noticed at the same lower levels (2 g L^−1^), with a notable synergism value of 8.6. Moreover, the combination of these inhibitors in a ratio of 1 : 1 at lower levels significantly boosted the performance to 93%.^58^

**Table 5:** Characteristics of some representative examples of synergistic between corrosion inhibitors

Inhibitor	Environment	Conc.	Method of corrosion monitoring	IE %	Ref.
Sodium nitrite	Carbon steel	2 g L^−1^		65%	58
Sodium nitrite		2 g L^−1^		53%	
Nitrite + molybdate		<2 g L^−1^		93%	
Sodium tungstate + ZnSO_4_	Mild steel/natural seawater	1000 ppm + 300 ppm ZnSO_4_	WL, EIS, PDP, XPS	84.81	66
Zinc aluminium molybdenum orthophosphate hydrate (ZAM)/zinc calcium strontium aluminium orthophosphate silicate hydrate(ZCP)	Mild steel/NaCL	1 g each of ZAM and ZCP	EIS, PDP, electrochemical noise, SEM, EDS	92%	276
Sodium nitrite	Mild steel/3.5NaCL	0.5 M NaNO_2_	WL	10&	277
Potassium chromate		0.5 M K_2_CrO_4_		33%	
Potassium chromate/sodium nitrite		0.5 M NaNO_2_ + 0.5 M		56%	
		K_2_CrO_4_			
Phenylalanine (PA) + Zn^2+^ ions	Carbon steel/well water	5 ppm Zn^2+^ and PA 150 ppm	WL. PDP. EIS. SEM-EDX.	90%	265
Glutamic acid with Zn^2+^	Carbon steel/sea water	200 ppm glutamic acid with 25 ppm Zn^2+^	WL, EIS, PDP, AFM, SEM	87%	258
Zn^2+/^sodium salt of phenyl phosphonic acid (PPA)	Neutral solution containing 60 ppm Cl^−^	300 ppm PPA: 50 ppm Zn^2^		95%	278
Chitosan + KI	Mild steel/15% H_2_SO_4_	5 g CH + 5 mM KI	WL, DEIS, PDP, AFM, SEM and EDS	97.6	196
Sodium lignosulfonate-zinc acetate (SLZA)	Mild steel/3.5NaCL	500 ppm sodium lignosulfonate + 300 ppm zinc acetate	WL, EIS, PDP, XPS, FTIR, SEM	96%	279
Polyaspartic acid (PASP)	Mild steel/3.0 NaCL	2000 ppm PASP	WL, EIS, PDP, XPS, FTIR, SEM, AFM, MD, QC	56%	45
PASP + Zn^2+^		500 ppm PASP + 1 ppm Zn^2+^		97%	
Xanthan gum (XG)	Mild steel/1 M HCl	1000 ppm	WL, EIS, PDP, SEM	74.8%	208
Xanthan gum (XG) + SDS		1000 + 5		83.17%	
Xanthan gum (XG) + CPC		1000 + 5		75.89%	
Xanthan gum (XG) + TX		1000 + 5		82.31%	
Chondroitin sulfate derived from pig cartilage (CS-PC) sodium alginate (SA) CS-PC + SA	Mild steel/1 M HCl	600 ppm	MD, PDP, EIS QC, WL	73.93%	212
		400 ppm		69.03%	
		400 + 400 ppm		96.88	
Tannic acid (TA)	Mild steel/1 M HCl	1.0 g L^−1^	PDP, EIS, SEM, QC	91%2	280
Gallic acid (GA)		1.0 g L^−1^		74.5%	
TA + GA		0.9 g L^−1^ TA^−1^: 0.1 g L^−1^ GA^−1^		93.3%	

### 4.2 Synergistic impact between organic and inorganic substances

**Fig. 8:**
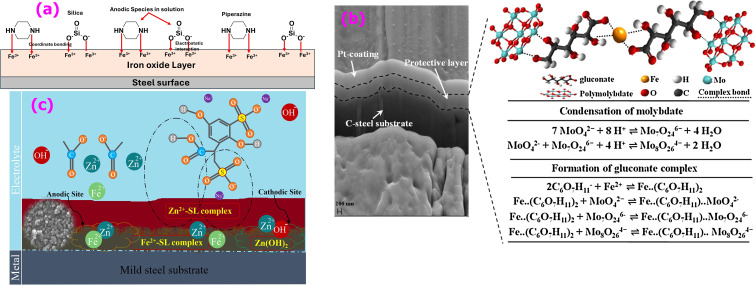
Schematics of synergistic effects of mixtures of (a) piperazine and silicate, compiled from ref. 288 (b) gluconate and molybdate, reprinted with the permission of ref. 289, copyright 2024; and (c) zinc acetate and sodium lignosulfonate (SL), reprinted with the permission of ref. 279, copyright 2024.

The widespread use of organic inhibitors is attributed to their eco-friendly features, cost-effectiveness, and ability to be employed in smaller quantities compared to inorganic inhibitors. As a result, these inhibitors have become the primary focus of research in various fields. However, the effectiveness of a single organic material is significantly impacted by factors such as the temperature, condition of the metal surface and its surrounding medium, and immersion time. In certain specialized industries, a sole organic agent may not meet the stringent requirements for corrosion prevention. To address this issue, combining organic and inorganic inhibitors, as well as incorporating organic inhibitors with trace cations, alkaline earth salts, halides, or other anions, can significantly boost the anticorrosion performance and stability of the targeted system, while also minimizing the overall usage of inhibitors.^285,286^ As an example, the synergistic mechanism of sodium tungstate and a Mannich base (C_15_H_15_NO) was examined.^287^ Their research revealed that the Mannich base initially attaches to the targeted surface, creating a film owing to its robust adsorption energy. Following this, tungstate ions are incorporated into the defects within the created layer by Mannich base, resulting in a tightly sealed film, as well as forming hydrogen bonds with hydrogen ions, which effectively block corrosive ions from penetrating the adsorption film. This process significantly minimized the existence of corrosive ions near the employed surfaces. In a research, the synergistic impact of sodium silicate and piperazine inhibitors on ST-14 steel was explored.^288^ The findings revealed that the combination of these two inhibitors greatly promoted the steel's resistance against corrosion, as demonstrated by PDP and EIS measurements, with improvements of approximately 87% and 76%, respectively. The most effective corrosion inhibition was achieved with a combination of 10–15 ppm sodium silicate and 2 ppm piperazine (PIP). Observing the interaction between the iron oxide layer and inhibitor molecules, it can be inferred that physical adsorption played a more prominent role in the film generation for both PIP and sodium silicate inhibitors. The researchers suggested that oxygen atoms of silica acted as bridges linking piperazine to metal ions at the surface defects. This connection enables the formation of a thicker and more impenetrable film at the anodic sites (as shown in Fig. 8a). Another study showed that the combination of sodium molybdate and benzotriazole (BTA) resulted in a protective layer consisting primarily of BTA-Fe and FeMoO_4_.^290^ This structure enhanced the density of the FeMoO_4_ corrosion inhibition film and facilitated the conversion of FeOOH into a stable Fe_2_O_3_ compound. Additionally, when the pH levels are maintained between 8.0–10.0, the inhibition performance reached 99%, and the system exhibited high stability and required a low concentration for optimal effectiveness. Furthermore, an inhibitor composed of gluconate, as well as small quantities of molybdate was reported.^289^ The synergistic impact between gluconate and molybdate was elaborated (Fig. 8b). PDP measurements revealed that the existence of gluconate made the corrosion of tested metal kinetically and thermodynamically unfavorable, surpassing the impact of molybdate alone. The SEM, FTIR, and XPS analysis revealed that gluconate acted as a bridge between iron and molybdate, leading to the formation of a protective layer that hindered corrosion, as shown in Fig. 6b.

Numerous studies have confirmed that the inclusion of transition metal cations can greatly boost the performance of organic corrosion inhibitors. For instance, the synergistic features of sodium lignosulfonate–zinc acetate (SLZA) in hindering corrosion of employed MS in a 3.5 wt% NaCl was examined.^279^ The findings from electrochemical data demonstrated that the joint action of ZA and SL effectively hindered uniform corrosion. The synergistic impact was quantified to be approximately 9, and the overall resistance exceeded 400 kΩ cm^2^ when both SL and ZA were employed. Moreover, the SLZA system exhibited an impressive inhibition of around 96%. Based on SEM-EDS and XPS analyses, the researchers proposed that the film composed of zinc-containing compounds and SL-based complexes played a critical role in hindering corrosion, as shown in Fig. 8c. In another research, the effectiveness of PASP as an inhibitor was significantly boosted by the inclusion of zinc ions, reaching a performance of 97% at a level of 0.5 g L^−1^. By adding Zn^2+^ the performance of polyaspartic acid was noticeably boosted as it hindered the cathodic sites of the localized corrosion cells.^45^ The zinc ions were found to adhere to the surface, replacing Fe^+^ ions. This led to the inducement of a PASP–Zn complex, which created a protective layer and effectively hindered corrosion. Analysis using AFM and EDX revealed the formation of a thick layer following the inclusion of zinc ions.^45^

Halide ions, especially iodide ions, have been utilized to enhance the performance of organic corrosion inhibitors. The order of synergistic impacts typically follows: Cl < Br < I, where I^−^ ions exhibit the best synergistic impact, thanks to their larger size (216 pm) and ease of polarizability. The robust electronegativity of halides allows for the creation of bridges between the metal surface and the positive end of the employed inhibitor. This connection helps to extend their surface coverage, resulting in better protection against corrosion. For instance, The effectiveness of CS and KI additive in inhibiting St37 steel corrosion, in a 15% H_2_SO_4_ medium was examined.^196^ The addition of KI significantly augmented the CS inhibitory performance, reaching 92%. PDP and EIS analysis revealed that the CS–KI film was more robust and reflected boosted effectiveness over longer immersion periods. The inhibitory performance of CS declined at elevated temperatures, while the CS–KI combination reflected a boosted trend and achieved its best inhibition of 99.72% at 60 °C. A calculated synergism emphasized that the boosted performance of CS–KI was a result of a synergistic effect.^196^ Furthermore, the impact of KI on tannin anticorrosion performance was examined.^291^ The findings reflected that adding just 0.025% KI to the tannin solution minimized the anodic current density (*I*_corr_) as revealed by electrochemical tests and reflected improved corrosion inhibition performance.

### 4.3 Synergism between organic inhibitors

Numerous investigations have been conducted to look at how combining organic inhibitors impacts the effectiveness of inhibition. In one study, 1000 ppm xanthan gum (XG) exhibited a 74.24% inhibition; however, the addition of low levels of surfactants, namely Triton X-100 (TX), cetyl pyridinium chloride (CPC), and sodium dodecyl sulfate (SDS), marginally boosted the corrosion inhibition efficiency.^208^ UV-visible analysis emphasized the creation of a complex between Fe^2+^ and XG. Additionally, SEM images reflected distinct morphological changes in the presence of XG and XG-surfactant additives. Moreover, an inhibitor consisting of a combination of sodium alginate (SA) and chondroitin sulfate obtained from pig cartilage (CSPC) was developed.^212^ The synergistic impact of these two polysaccharides on corrosion inhibition under 1 M HCl was investigated. The results suggest that the CSPC and SA mixture exhibit a robust impact, outperforming individual inhibitors (with performance of 95.18% compared to 72.78%). Furthermore, quantum mechanical calculations demonstrated that the bond creation between tested metal and tested inhibitors occurred *via* charge transfer between the CSPC, SA and iron with evidence of partial retro-donation bonding type. Furthermore, the synergism of a combination of Anacardium occidentale (cashew gum) and Acacia Senegal (Arabic gum) in corrosion protection of MS study was elaborated.^292^ The inhibitor adsorption followed the Langmuir isotherm, reflecting a chemisorption behavior between the metal surface and the gums. This was emphasized by the values of the free Δ*G* of −16.47 and −15.61 kJ mol^−1^ at 303 K and 333 K, respectively. Dipole moment, molecular weight, and *E*_Homo_ were found to affect the gum mixture binding energy, and GC-MS was used to assess the gum constituent's hydrophobicity, which was shown to be a contributing factor to its efficacy as an inhibitor in acidic environments.^292^

In summary, mixed corrosion inhibitors are a suitable choice when strict corrosion inhibition performance is needed in challenging processing environments. Among the available options, organic/organic systems offer the advantage of being non-toxic and biodegradable, making them environmentally preferable over organic/inorganic and inorganic/inorganic systems. However, it is crucial to mention that even though the amounts of inorganic components used in these mixtures is small, they have the potential to bioaccumulate to dangerous levels over time.

### 4.4 Synergistic corrosion-inhibition mechanisms

Synergistic corrosion inhibition mechanisms exhibited by inhibitors combinations can work *via*, the gaps-filling, bond formation between different inhibitors, cooperative and complementary adsorption, and mutual interactions of inhibitors in the bulk solution. The gaps-filling approach stipulates that different inhibitor molecules act in a complementary manner, where one type of inhibitor component fills the gaps and defects within the protective film established by the other inhibitor, thereby synergistically enhancing the overall corrosion protection. The process starts with the adsorption of the organic inhibitor compound, such as Mannich bases or polysaccharides, onto the metal surface to form a primary adsorption layer, which serves as the foundation for the development of the protective film. In a later stage, the inorganic inhibitors begin to incorporate or bind into the organic inhibitor layer while filling the gaps and defects present within the organic film, creating a more uniform and complete surface coverage.

Furthermore, the inorganic inhibitor can interact with the organic inhibitor film, leading to the formation of complex metal–organic compounds. These complex compounds contribute to the enhanced stability and protective properties of the passive layer, making it more compact, dense, and resistant to the penetration of corrosive species. The complementary adsorption and filling of the film by various inhibitor components create a synergistic effect, wherein the weaknesses or deficiencies of one inhibitor type are compensated by the strengths of the other.

On the other hand, cooperative adsorption is another key mechanism for the synergistic effect of mixed organic corrosion inhibitors. In this mechanism, the two inhibitors adsorb on the metal surface in a sequential manner, where one inhibitor first chemisorbs onto the surface, creating a foundation for the second inhibitor to then adsorb on top of the initial layer. This cooperative adsorption can lead to the formation of a more compact, stable, and protective film on the metal surface, resulting in enhanced corrosion inhibition. The synergistic effect arises from the combined protective action of the two inhibitors, where they work in harmony to provide better coverage and protection compared to individual inhibitors.

However, complementary adsorption is another prospective mechanism for the synergistic inhibition effect, where the two inhibitors may preferentially adsorb on different sites of the metal surface, effectively covering a larger area and providing more comprehensive protection. For instance, one inhibitor may predominantly adsorb on the anodic sites, while the other inhibitor adsorbs on the cathodic sites, leading to the inhibition of both the anodic and cathodic corrosion processes. Furthermore, inhibitors mutual interactions in the bulk solution can contribute to the synergistic inhibition effect. These mutual interactions may include the formation of complex species or micelles, which can enhance the transport and adsorption of the inhibitors onto the metal surface. This improved availability and adsorption of the inhibitors on the metal surface can result in a synergistic inhibition of corrosion. Another mechanism of synergistic inhibition involves the cooperative adsorption of the mixed inhibitors on the metal surface. The adsorption of one inhibitor can modify the surface properties, creating more favorable sites for the adsorption of the other inhibitor. Additionally, the presence of multiple inhibitors can promote the formation of insoluble metal–inhibitor complexes on the metal surface. These complex species can act as a physical barrier, blocking the access of corrosive species to the metal surface and effectively inhibiting the corrosion process. In some cases, the synergistic effect may involve the combined action of anodic and cathodic inhibitors.

In summary, synergistic corrosion inhibition results in a more robust and impermeable protective barrier against corrosion, making the hybrid corrosion inhibitor system particularly ideal for harsh processing environments where strict corrosion inhibition performance is required. In addition to mutual interactions between inhibitors in the bulk solution that promote the inhibitors diffusion towards the metal surface, the underlying mechanisms governing the synergistic effect of hybrid/composite corrosion inhibitors involve complex interactions at the molecular level, such as gaps-filling, cooperative and complementary adsorptions, impermeable film formation, and the creation of inorganic–organic and organic–organic inhibitors' bonding. The nature and concentration of the inhibitors, the metal–environment system, and other variables can all affect the precise mechanism underlying the synergistic impact in mixed corrosion inhibitor systems. However, in most cases, multiple mechanisms work cooperatively.

## 5. Inhibition performance validation

### 5.1 Weight loss

The corrosion inhibitor's performance is a critical aspect of corrosion mitigation strategies.^293–295^ Weight loss (WL) methods are commonly utilized to determine the effectiveness of corrosion inhibitors in various environments. WL analysis is a relatively simple and cost-effective method compared to other advanced techniques and provides both a quantitative measure of the corrosion rate, and a realistic simulation of the actual corrosion conditions in a particular application. The WL method primarily involves exposing metal specimens to the corrosive environment under controlled conditions over a specific period.^45,293^ The typical procedure involves a series of steps for the preparation and analysis of the metal sample. Initially, the sample is sanded employing emery paper to prepare it for the experiment. After that, it is dried, washed with double distilled water, and degreased with acetone. Scale with ±0.01 mg sensitivity is employed to estimate the specimen's weight for the measurement. The recommended procedure calls for submerging the metal sample in various test solutions for a certain amount of time at a predetermined temperature. This is done with and without different concentrations of inhibitors. Following the experiment, the material is dried, cleaned, and weighed one more time.^294^ To ensure precision, the experiments should be executed in triplicate, and the average values should be considered. The inhibition efficiency *η*_w_ (%), surface coverage (*θ*), and corrosion rate (Cr) can be calculated using the following equations:^296,297^
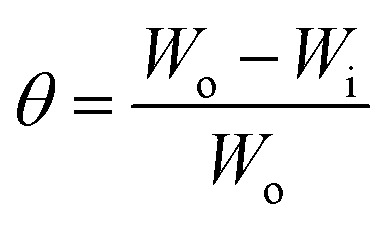

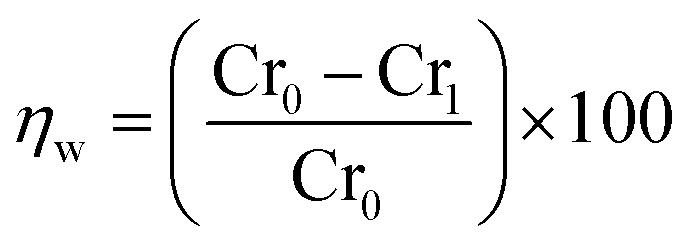

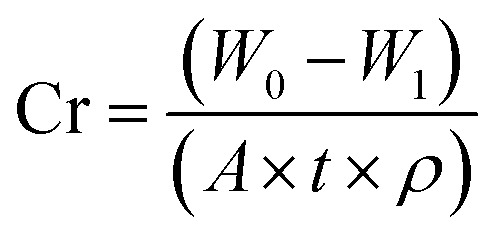


For instance, the WL method was used to assess the corrosion rate of MS under acidic conditions, in the presence and absence of the corrosion inhibitor.^293^ Notably, they achieved a high inhibition efficiency of 89.5% when using 400 mg L^−1^ inhibitor. In another study, WL monitored the inhibition performance of zinc ions and PASP at varying concentrations, where the addition of 2 mg L^−1^ zinc ions minimized WL compared to the use of PASP alone.^45^ Moreover, another investigation explored the impact of temperature on inhibition efficacy using the WL approach.^295^ The study observed that higher temperatures minimized the adsorption of PESA onto the examined metal surfaces. Consequently, this resulted in minimized surface coverage and subsequently lowered efficiency. Similarly, because of severe etching and degradation or desorption of inhibitor molecules, higher temperatures caused a reduction in surface coverage.^298^

Overall, the WL method plays a crucial role in evaluating the performance of corrosion inhibitors. It provides valuable insights into the effectiveness of inhibitors under specific conditions, aiding in the design and optimization of corrosion protection strategies for various industrial applications.

### 5.2 Surface analysis

SEM is a powerful imaging technique that provides high-resolution images of the sample surface.^299–301^ It utilizes a focused electron beam to scan the sample, generating secondary electrons, backscattered electrons, and characteristic X-rays.^286,302–304^ The secondary electron images obtained from SEM provide detailed information about the surface morphology, such as surface roughness, the presence of cracks, pits, or other corrosion features.^305–307^ Additionally, backscattered electron imaging can reveal compositional variations on the surface, highlighting the presence of corrosion products or the distribution of the inhibitor.^302^ EDX is used in conjunction with SEM to provide elemental analysis of the sample.^308–311^ It detects characteristic X-rays emitted by the atoms in the sample when excited by the electron beam. In the context of evaluating corrosion inhibitors, EDX can help determine the effectiveness of the inhibitor in reducing the concentration or presence of corrosive elements, such as chloride ions or oxygen.^312,313^ By comparing the elemental composition of metal surfaces with and without the inhibitor, changes in corrosion product formation or inhibitor adsorption can be assessed.^314,315^

**Fig. 9:**
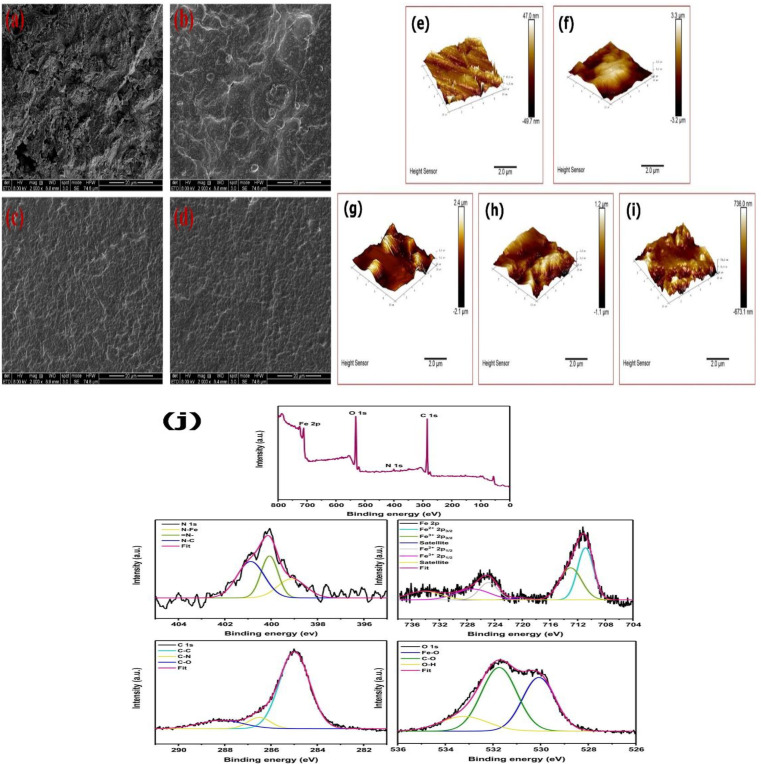
SEM images of mild steel (a) 1 M HCl, (b) chitosan, (c) chitosan-5-HMF1 and (d) chitosan-5-HMF3, reprinted with the permission of ref. 318, copyright 2024, Elsevier. AFM images of mild steel surface: (e) polished, (f) blank, in presence of; (g) SA, (h) SA-g-PMMA/Fe_3_O_4_, (i) SA-g-PMMA/TiO_2_,^183^ (j) XPS wide scan and high-resolutionscans of Fe 2p, C 1s, O 1s, and N 1s for MS in the 15% HCl solution containing 50 μM of CHEC, reprinted with the permission of ref ^283^, copyright 2024, Elsevier.

For instance, SEM images of steel submerged in the solution without corrosion inhibitors in simulated concrete pores with chlorine revealed many pits on steel surface, revealing that the steel has been severely degraded by the Cl^−^ present.^316^ Similarly, SEM pictures of the steel reveal signs of severe corrosion and comparatively extensive surface fractures.^317^ The majority of surface flaws were the sites where corrosion started, resulting in the production of corrosion products that most likely covered specific localized regions of the whole material surface. These corrosion byproducts created a porous layer that encouraged more corrosion, leading to a serious assault with surface fractures and long void.^317^ Furthermore, the formation of an inhibitory film on MS surface and the thickening of this film were confirmed by SEM images, which reflected the adsorption of PASP and its interaction with zinc ions.^45^ Another research also utilized SEM images to analyze the effect of chitosan-5-HMF on the MS surface after exposure to 1 M HCl, as illustrated in Fig. 9a–d.^318^ Without any inhibitors, the surface of MS exhibited uneven damage, characterized by the formation of pits of various sizes. Further, significant alterations were noted when mild steel was placed in a solution containing 200 mg L^−1^ of chitosan, as shown in Fig. 7b. Notably, the formation of pits and voids was eliminated. Conversely, when using the inhibitors chitosan-5-HMF1 and chitosan-5-HMF3 (Fig. 7c and d), the surface of the matrix became notably smoother and improved. This suggests that the adsorption of chitosan-5-HMF species hindered the contact between mild steel and the corrosive electrolyte, resulting in improved corrosion resistance. This inhibitory effect is further evidenced in the contact angle images. In another study, an EDX mapping examination revealed a significant presence of Cl in the affected area owing to its corrosive activity.^319^ Conversely, a reduction in chloride signal owing to the generation of a protective layer on the MS was observed and this protective layer effectively hindered chloride ions from reaching the active sites of the MS.^317^ Moreover, the proportion of oxygen atoms in the blank solution, which corresponds to the rate of oxide formation on the MS surface, was found to be 10.07%.^320^ However, following the inhibitor's addition, this ratio minimized significantly to 2.87% owing to the protective nature of adsorbed octacalcium phosphate (OCP) inhibitor molecules.

To gain quantitative insights into the surface morphology of metals, the utilization of AFM is necessary.^216^ AFM allows for the examination of utilized specimen topography in a 3D fashion. By employing AFM, one can evaluate the surface topography of a sample and observe shifts in roughness resulting from corrosion or the presence of inhibitors.^321^ For both protected and unprotected samples, the average roughness values, expressed in nanometers and root mean square (RMS) are calculated and compared.^322^ The quantitative investigation demonstrates that the maximum peak-to-peak height values and the RMS roughness of the inhibitor-coated metal surface are smaller than those of the untreated metal surface.^323,324^ For instance, the initial roughness (Ra) measurement of polished mild steel was 7.96 nm (Fig. 9e). However, immersion in a 15% HCl solution without any inhibitor for 24 hours resulted in a significant increase in Ra to 973 nm (Fig. 9f). In the presence of SA, SA-g-PMMA/Fe_3_O_4_, and SA-g-PMMA/TiO_2_, the Ra values were measured at 414 nm, 282 nm, and 144 nm, respectively (Fig. 9g–i). Based on these findings, the SA-g-PMMA/TiO_2_ proved to be the most effective inhibitor, as it produced the smoothest surface. This suggests the formation of a protective layer or adsorption film on the metal surface, highlighting its superior inhibitory properties compared to the other inhibitors examined.^216^ Similarly, it was found that the MS that corroded in soft water had a rough, non-uniform appearance with big, deep holes; however, the MS surface was smoothed up after adding the inhibitor.^297^

XPS is a powerful approach employed in evaluating corrosion and corrosion inhibitors. When a high-energy X-ray beam is directed at a material's surface, photoelectrons are released, which is how XPS analysis works. These photoelectrons carry data about the elemental composition and chemical states of the atoms near the surface.^301^ XPS is useful in determining the chemical composition of the corrosion byproducts that are generated.^325,326^ XPS can also provide information about the chemical states of these elements.^146^ For example, iron may exist in different oxidation states as Fe(0), Fe(ii), or Fe(iii) species.^327,328^ By analyzing the binding energy of the iron photoelectron peaks, one can determine the oxidation state and the presence of different iron species on the corroding surface. By comparing the XPS spectra of inhibited and uninhibited surfaces, differences in the chemical composition and bonding states of the elements can be observed. For example, the presence of a corrosion inhibitor may result in the formation of a protective layer, which is evident from the XPS spectra showing new peaks or shifts in the binding energies associated with oxygen or other relevant elements.^329,330^ To confirm the adsorption of chemically modified hydroxyethylcellulose (CHEC) on the electrode surface, XPS analysis was conducted.^331^Fig. 9j displays the high-resolution plots and broad scan. The O and C bands exhibited a significant exsitance in the MS specimen immersed in the 15% HCl medium with 50 μM CHEC, which is expected as they are major constituents of the inhibitor structure. The O 1s peaks at 531.74 eV and 533.22 eV correspond to the C–O and C–OH groups, respectively, that are present in the CHEC structure. The C 1s peak at 288.15 eV indicates the presence of C–O, while it is located at 285.89 eV in the uninhibited systems. The binding of inhibitor molecules on the metal surface is responsible for the C 1s peak's shift towards higher energy. Furthermore, XPS examination of the CHEC system indicates the existence of N at 399 eV, which supports interactions between carbon (C), iron (Fe), and N. These results provide compelling evidence for the efficient adsorption of CHEC molecules onto the steel surface, which blocks active sites and inhibits corrosion. The nitrogen heteroatom and the accessible orbitals in the iron's atomic structure share electron pairs during the chemical adsorption process.^331^ Furthermore, XPS demonstrated that the adsorption of various gum-ased inhibitors on MS surface involved both chemisorption and physisorption.^294^ Similarly, the XPS analysis supports the conclusion that the adsorption of GAMo, GAMau, and GASe on the MS surface involves both physisorption and chemisorption.^294^ The XPS results provide strong evidence for the effective adsorption and outstanding inhibition properties of chitosan Schiff base (CS-FGA) molecules on the M.S. surface. The N 1s spectra clearly exhibit two distinct adsorption peaks, with the peak at 399.1 eV indicating the presence of N–Fe bonds. This finding further confirms the chemical adsorption between M.S. and CS-FGA.^39^

### 5.3 Electrochemical analysis

#### 5.3.1. Potentiodynamic polarization analysis

Potentiodynamic polarization (PDP) plots, commonly referred to as Tafel curves, serve as a vital tool in assessing corrosion rates and studying inhibition mechanisms. An effective approach in corrosion analysis involves the rate of cathodic reduction and anodic oxidation reactions may be ascertained using these graphs. The equivalent current density may be found by prolonging the linear portions of these curves until they meet. Utilizing the Tafel extrapolation method, crucial parameters such as cathodic Tafel slope (*β*_c_), corrosion potential (*E*_corr_), anodic Tafel slope (*β*_a_), and corrosion current density (*I*_corr_) can be derived. These parameters provide crucial insights into the nature of corrosion and aid in the analysis of inhibition mechanisms. When a corrosion inhibitor is present, it alters the polarization behavior of the metal electrode. In the case of an effective inhibitor, the polarization curves obtained in the presence of the inhibitor will exhibit a shift towards more positive (less active) potentials compared to the curves obtained without the inhibitor. This shift indicates a reduction in the corrosion rate and an increase in the protection efficiency. For instance, the impact of different concentrations of a specific PIL (imidazolium-derived polymeric ionic liquid) inhibitor on the corrosive behavior of MS specimens was examined under an acidic medium.^332^ Tafel plots were used to analyze the cathodic and anodic current densities in the absence and presence of the PIL. The findings reflected a notable decline in both current densities as the concentration of the PIL inhibitor rose. This suggests that the PIL inhibitor can influence both cathodic and anodic reactions, thus affecting the overall corrosion process. Furthermore, the *E*_corr_ changed to greater negative values in response to the PIL inhibitor's dominant cathodic inhibition. In another investigation, the addition of PASP to a NaCl solution caused the corrosion potential to move to the positive side. Thus, PASP essentially inhibits the anodic process meanwhile doesn't significantly affect the cathodic reaction.^45^ Similarly, the utilization of an AS inhibitor results in a significant change in the corrosion potential values (*E*_corr_), causing them to shift in a positive direction towards nobility.^333^ In a separate investigation, the analysis of PDP data demonstrated that the introduction of Chitosan-acrylic acid-poly succinimide terpolymer caused a shift in *I*_corr_ values to lower levels, demonstrating the inhibition of carbon steel corrosion by the existence of CTS-AA-PSI. At higher levels (500 mg L^−1^), the increased amount of CTS-AA-PSI molecules resulted in enhanced coverage of the carbon steel surface, leading to a robust inhibition performance (77.84%). Furthermore, higher dosages of CTS-AA-PSI were noticed to shift *E*_corr_ towards more positive values and greatly minimize the corrosion rate. During this process, the value of *β*_c_ gradually minimized from 398.3 mV dec^−1^ (blank) to 79.2 mV dec−1 (500 mg L^−1^), while *β*_a_ minimized from 81.7 mV dec^−1^ (blank) to 50.1 mV dec^−1^ (500 mg L^−1^). Importantly, the difference in Δ*E*_corr_ between the uninhibited and inhibited samples was less than 85 mV, demonstrating that CTS-AA-PSI predominantly served as a mixed-kind agent.^334^ Fig. S4† illustrates a remarkable decline in the current densities when sucrose derivative corrosion inhibitor (CHEC) is present at all temperatures. Additionally, the *E*_corr_ movement according to the medium temperature reflects the significant influence of temperature on the inhibitory effect of CHEC.^331^

#### 5.3.2. Electrochemical impedance spectroscopic analysis

EIS examines the electrical features of a material by applying alternating current (AC) signals and a range of frequencies are utilized to obtain a spectrum.^335,336^ Typically, EIS assessments are carried out using a standard three-electrode electrochemical cell setup. This setup consists of the working electrode (WE), counter or supplementary electrode (CE), and reference electrode (RE). The WE represents the metal sample under test.

Impedance spectroscopy is employed to determine the double-layer capacitance (*C*_dl_) and the resistance to charge transfer (*R*_ct_).^337^ The inhibition performance can then be estimated using these parameters. Effective adsorption of the inhibitor is demonstrated by an enhancement in *R*_ct_ and a minimization in *C*_dl_ as the inhibitor amount is increased.^73^ The impedance modulus (|*Z*|) represents the overall resistance to the electrochemical processes occurring at the metal–solution interface, and a higher |*Z*| value indicates better corrosion inhibition. The phase angle (*θ*) provides information about the nature of the electrochemical processes, such as the presence of capacitive or inductive behavior. The EIS data is typically presented in the form of Nyquist and Bode plots, which provide a visual representation of the electrochemical impedance characteristics. The Nyquist plot shows the imaginary component of the impedance (*Z*′′) *versus* the real component (*Z*′), while the Bode plot shows the logarithm of the impedance modulus (|*Z*|) and the phase angle (*θ*) as a function of the logarithm of the frequency. By comparing the EIS data between the uninhibited and inhibited systems, the effectiveness of the corrosion inhibitor can be evaluated. Typically, the *R*_ct_ value increases, the *C*_dl_ value decreases, and the |*Z*| value increases in the presence of an effective corrosion inhibitor, indicating an improvement in the corrosion inhibition performance.

To further analyze the EIS data and gain a better understanding of the corrosion inhibition mechanism, an equivalent circuit model can be employed. The choice of the equivalent circuit model depends on the specific system and the processes occurring at the metal–solution interface. The equivalent circuit model consists of various electrical elements, such as resistors, capacitors, and inductors, which represent the different electrochemical processes and the characteristics of the inhibitor film. By fitting the experimental EIS data to the equivalent circuit model, the values of the circuit elements can be obtained, providing insights into the inhibition mechanism and the changes in the interfacial properties due to the presence of the corrosion inhibitor. Overall, the EIS analysis together with the Nyquist and Bode plots, and the use of equivalent circuit modeling, is a powerful tool for evaluating the performance and understanding the mechanisms of corrosion inhibitors.

For instance, as shown in Fig. S5a and b,† EIS was utilized to examine the electrochemical features of the MS surface in both blank and Cs-g-l-arginine containing solutions.^338^ The Nyquist plot demonstrated a semicircle, with greater diameters noticed in the presence of Cs-g-l-arginine compared to the blank solution. Additionally, the size of the semicircle increased as the inhibitor's concentration rose. This demonstrates that Cs-g-l-arginine exhibits significant resistance to charge movement compared to the unprotected MS surface in the blank solution, attributed to its adsorption onto the Cs-g-l-arginine surface.^338^

### 5.4 Computational studies

Unlike the time consuming and equipment-intensive nature of experimental measurements, computational techniques provide a versatile and efficient means of evaluating the effectiveness of inhibitors. By analyzing the structural properties of corrosion inhibitors, computational approaches offer valuable insights into their potential impact. This predictive capability is particularly advantageous as it allows for the assessment of organic molecules' corrosion-inhibiting performance even before conducting time-consuming and resource-intensive experimental tests. The power of computational analysis lies in its ability to facilitate the design and development of efficient inhibitors. Through the utilization of computer software, specifically density functional theory (DFT), researchers can estimate reactivity indices and establish quantitative structure–activity relationships (QSAR). This allows for a systematic understanding of how different molecular structures correlate with inhibitor performance. Moreover, atomistic, and molecular simulations, including Monte Carlo (MC) and Molecular Dynamics (MD) simulations play a vital role in providing detailed insights. These simulations provide a deeper understanding of the likely orientations of inhibitor adsorption on the metal surface under investigation. By visualizing these interactions at the atomic and molecular levels, researchers can gain crucial information to guide the design of corrosion inhibitors with enhanced effectiveness.

#### 5.4.1. Quantum mechanical calculations

A popular computer modeling approach to analyzing chemical characteristics, chemical processes, electronic structure, and charge distribution, is called density functional theory (DFT).^13,175,339^ The frontier molecular characteristics of inhibitor molecules, namely the highest occupied molecular orbital (HOMO), the energy gap (Δ*E*_gap_), and the lowest unoccupied molecular orbital (LUMO), are intimately related to their capacity to adsorb to metallic surfaces. DFT calculations offer valuable quantum chemical features such as *E*_HOMO_, *E*_LUMO_, electron density, Δ*E*_gap_, electrostatic potential (ESP), and the quantity of migrated electrons (Δ*N*). Determining Δ*N* involves employing Koopman's theorem, which relies on several parameters including electron affinity (*A*), chemical softness (*σ*), absolute chemical hardness (*η*), electronegativity (χ).^340^ Electrons tend to migrate from a system with lower electronegativity (*χ*) to one with higher electronegativity until a condition of equilibrium is reached when considering electronegativity.^341^ An essential aspect when evaluating the stability and reactivity of molecules is absolute hardness (*η*), as hard molecules were shown to have a significant energy gap and a limited electron-donating ability. Conversely, soft molecules possess a small energy gap and a significant ability to donate electrons, thus making them more reactive compared to their harder counterparts. Moreover, an increased dipole moment strengthens the adsorption of the inhibitor onto the metal surface, thus facilitating the process of inhibition.^341^ Therefore, highly effective inhibition mechanisms generally exhibit elevated softness, *E*_HOMO_, and dipole moment values, alongside lower electronegativity, *E*_LUMO_, and hardness values.^342^ For instance, sodium oleate (SO), and calcium lignosulfonate (CLS) were evaluated as corrosion inhibitors.^343^ The sulfonate groups of the CLS molecule, which functioned as electron donors, were the main contributors to the HOMO and LUMO distributions at the end of the chain, according to quantum chemical analysis. On the other hand, sites for electron acceptance were supplied by the lignin functional group, sinapyl alcohol monomer. The lengthy carbon chain's center and the chain end of the oleic acid group were home to the SO-inhibitor molecule's HOMO and LUMO, respectively. According to the *E*_HOMO_ and *E*_LUMO_ values, CLS outperformed SO inhibitor.^343^ In another investigation, the TTA inhibitor exhibited a dispersed concentration of electron density in the middle area of its structure, which included heteroatoms like O, S, and N.^344^ On the other hand, TTA's LUMO electron density is dispersed on both sides of the core triazole ring, suggesting that these particular molecular regions are in charge of absorbing electrons from the iron surface. The HOMO and LUMO electrons serve crucial roles in the electron donation and acceptance processes involved in the adsorption processes onto the Fe surface. The TTA inhibitor may adsorb onto the iron surface as evidenced by its strong ionization potential values and very weak electron affinity. The TTA inhibitor is predicted to have high adsorptive qualities on the Fe surface due to its high chemical softness and low hardness. Furthermore, TTA's important capacity to donate electrons to the Fe (110) surface is shown by the fraction of transferred electrons (Δ*N*) of TTA [Δ*N* (TTA) = 0.0502].^344^ Similarly, DFT calculations was employed for various type of *Boswellia serrata* gums namely xylose, arabinose, galactose, and glucuronic acid. The LUMO and HOMO isosurfaces were noticed to be distributed over the heteroatoms and rings, this reflects that these regions are the adherent sites for the inhibitor molecules.^341^ Positive values for quantum parameters imply that molecules are inclined to transmit electrons to the target surface. High inhibition and reactivity abilities of the molecules are shown by lower Δ*E* values as well. When compared to the remaining compounds, xylose demonstrated greater reactivity.^341^

#### 5.4.2. Molecular dynamics (MD)

Molecular dynamics (MD) is a tool that allows us to study the behavior of atoms and molecules over time. In the context of adsorption, MD modeling provides valuable insights by simulating the interactions among metal surface and adsorbate molecules. One important aspect that MD modeling can assess is the orientation of the molecules on the metal surface.^175,345,346^ How the molecules are positioned and aligned relative to the metal surface strongly influences their adsorption features. For instance, when an inhibitor molecule lies flat on the metal surface, it covers a larger region, promoting the chances of binding to active sites and granting a protective layer.^231,347,348^ In contrast, if the inhibitor molecule adopts a vertical or non-planar orientation, its coverage on the metal surface may be minimized, limiting its effectiveness as an adsorbate. In MD simulations, the term “adsorption energy,” which is also referred to as “interaction energy,” represents the amount of energy needed or released when a group of inhibitor molecules adhere to a metallic surface. This energy is crucial to understanding the underlying mechanism of adsorption. Several key factors, namely inhibitor valence, electronegativity, and reactive site coordination, are important variables in influencing the energy associated with adsorption.^349,350^ The estimation of energy can be expressed as follows:^351^*E*_interaction_ = *E*_total_ − (*E*_metal+solution_ + *E*_molecule_)where *E*_metal+solution_ signifies the energy of the cell in the absence of any retarding substance. On the other hand, *E*_molecule_ stands for the energy of the applied molecules of inhibitor when it exists on the surface. Lastly, *E*_total_ represents the collective energy of the simulated system. A negative energy demonstrates a spontaneous and stable adsorption operation. This reflects that the inhibitor molecules are attracted to the metal surface, initiating various chemical, physical, or combined adsorption forces. A more potent inhibitory performance is achieved through increased adsorption capacity, leading to a more substantial release of energy (illustrated by a larger negative value).^36^ The energy required to bind various systems consists of several factors including dissociation energy, electron binding energy, gravitational binding energy, and atomic binding energy. In metal surfaces, inhibitor molecules have a crucial impact on bond dissociation energy as chemical bonds are formed and broken constantly. The strength of the attraction between the inhibitor molecule and the metal has a direct effect on the interaction energy. A higher value signifies stronger bonding and increased inhibition efficiency. As a result, the *E*_binding_ can be calculated by taking the reciprocal of the *E*_interaction_ to determine its magnitude. MD simulations have found widespread application in exploring the interaction among surfaces and inhibitor molecules within various electrolytic environments. For instance, MD simulations extensively examined various surfactants-based inhibitors.^352^ The findings from these simulations reflect a notable attraction of the target surface to the benzene ring of inhibitor, with the ring often aligning parallel to the surface. This behavior can be reasonably explained by the interaction between π-electrons and the metal d-orbitals. Furthermore, the adsorption features of two variants of pyridine-carboxaldehyde thiosemicarbazone (PCT), specifically 2-PCT and 4-PCT, on mild MS was examined by MD simulations that showed that both 2-PCT and 4-PCT exhibited nearly horizontal direction during the adherence process.^353^ Furthermore, the π– and non-bonding electrons of the pyridine ring, along with the nitrogen and sulfur atoms of thiosemicarbazone, actively engaged in electron acceptance and a donation from the targeted metal surface. The estimated binding energies were determined to be 85.52 kcal mol^−1^ for 2-PCT and 83.39 kcal mol^−1^ for 4-PCT. Furthermore, an MD study examined the behavior of amphiphilic surfactant inhibitors and revealed that these molecules adhered to the metal surface in a specific arrangement, where the inhibitor's polar heads and hydrophobic tails alternate, resulting in the formation of a cohesive and uniform adherent layer.^354^ Consequently, this process gives rise to two distinct binding energies for nitrogen atoms. The first category of molecules showcases electrostatic interaction between the metal surface and the nitrogen atom (head orientation onto the surface). On the other hand, the second category of molecules experiences negligible surface interaction, as the head group is oriented towards the bulk medium. Furthermore, examined the adsorption patterns of 3-phenyl-1,2,4-triazole-5-thione (PTT) onto low-caron steel was examined, focusing on the Fe(110) surface, the most stable facet of this body-centered cubic metal.^355^ Two simulated media were employed: PTT/NaOH/Fe(110) related to a non-corrosive medium, and PTT/NaOH/NaCl/Fe(110) corresponding to a corrosive medium, as illustrated in Fig. S6a.† Analyzing the adsorption configurations of the studied molecule in both media reflected that it aligned horizontally to the metal surface, offering significant surface protection. Additionally, the investigation demonstrated that the existence of NaCl caused a decrease of around 10% in *E*_binding_. This reduction highlights the detrimental impact of Cl^−^ ions on the protective film of the molecule on the metal surface. Furthermore, MD study of the inhibitor's binding with MS in a 1 M HCl solution was performed.^351^ As shown in Fig. S6b,† the final arrangements of the *Lavandula Mairei* Humbert extract (LM) inhibitor are illustrated after achieving equilibrium at various simulated temperatures (303, 313, 323, and 333 K).^351^ The findings demonstrate that the LM inhibitor firmly attaches to the Fe(110) surface in a horizontally flat position, facilitated by the presence of oxygen atoms and aromatic rings within the inhibitor structure. As the temperature rises, there is a lowering in the negative interaction energy between the Fe(110) system and the LM inhibitor. This suggests that the bonding strength diminishes as the temperature rises. Further, MD simulations were employed to predict the adsorption behavior of the CHEC molecule on a Fe (110) surface in an aqueous environment. The simulations revealed that the CHEC molecule adopts a flat-lying adsorption orientation on the Fe surface, maximizing surface coverage. The binding energy (*E*_bind_) calculations showed a substantially negative value (−137.65 kJ mol^−1^), attributed to dispersive interactions from the π electron delocalization and the high polarizability of the amine N atoms. This adsorption orientation was found to be stable and consistent with experimental observations of strong corrosion inhibition performance. Additional simulations in the gas phase confirmed the orientation, showcasing the favorable interaction between the Fe (110) surface and the diphenyl and amine groups, which are significant in the adsorption process. These interactions are expected to remain intact even at elevated temperatures.^331^

### 5.5 Summaries of evaluation techniques

**Fig. 10:**
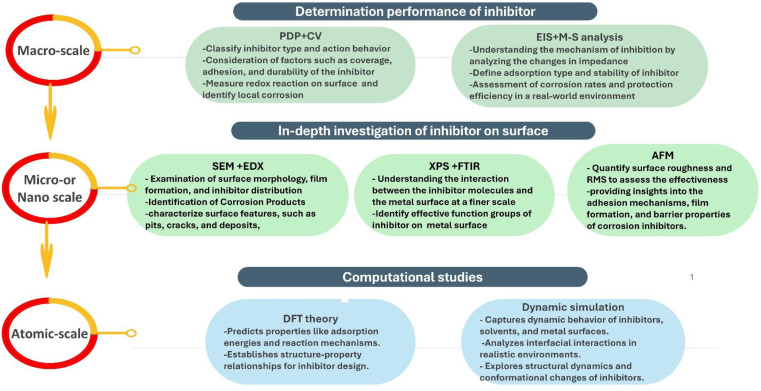
Summary of inhibition performance validation approaches.

Corrosion detection and monitoring are crucial in various industries to prevent infrastructure damage and equipment failure. Electrochemical techniques provide a non-destructive and fast way to assess corrosion and evaluate the effectiveness of inhibitors. However, challenges like electrode polarization, surface roughness, and corrosion by-products can hinder these techniques. Microscopy, including SEM and AFM, offers high-resolution imaging to examine corrosion products, pitting, and surface topography. Spectroscopic approaches like infrared spectroscopy and XPS provide detailed information on molecular composition and chemical changes on material surfaces, aiding in inhibitor characterization and corrosion product identification. Computational studies using DFT and MD simulations help understand atomic-level interactions between inhibitors and metal surfaces, predicting corrosion reactions' thermodynamics and kinetics. By combining electrochemical, microscopy, spectroscopic, and computational techniques, researchers and engineers can comprehensively understand corrosion processes. This multidisciplinary approach facilitates the development of more effective corrosion prevention strategies, optimization of inhibitor formulations, and informed material selection in various industries. Fig. 10. Illustrate summary of characterization techniques.

## 6. Comprehensive economic analysis of the corrosion inhibitor market

Corrosion poses a significant economic threat, resulting in substantial financial losses. According to the World Corrosion Organization, approximately 25% of steel production is impacted by corrosion yearly, equating to five tons per second or 150 million tons annually. Recent studies have shown that leading companies in the oil and gas sector are spending around $1.372 billion yearly to address corrosion-related issues. Ignoring the issue of corrosion can have significant financial consequences for various sectors. Even highly developed states and countries with advanced technology are impacted by corrosion and its substantial impacts. For example, in 1998, the USA incurred losses of around $276 billion as a result of corrosion-related effects, which accounted for approximately 3.1% of the gross domestic product. As of 2011, the overall estimated cost of corrosion exceeded $2.2 trillion, rising to $2.5 trillion by 2016, amounting to 3.4% of the global gross domestic product. In India, the yearly cost of corrosion is less than $100 billion, with South Africa reporting a direct cost of $9.6 billion.^25,26^ Implementing proper corrosion protection methods could potentially reduce these losses by 15–35%.^25,26^

The global corrosion inhibitor market is a dynamic and rapidly expanding industry fueled by the escalating demand for effective corrosion protection across a wide range of sectors. According to recent industry analyses, the global corrosion inhibitor market was valued at US$ 8.3 billion in 2022 and is expected to reach $13.34 billion by 2034, with a Compound Annual Growth Rate (CAGR) of 4.1% during the forecast period. This substantial market growth is driven by the increasing focus on corrosion prevention in vital infrastructure projects, the surge in demand for corrosion protection in the oil and gas sector, and the growing emphasis on sustainable and eco-friendly solutions.

**Fig. 11:**
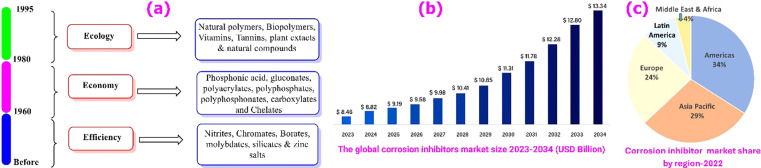
(a) Development of corrosion inhibitors over year, reprinted with the permission of ref. 356, copyright 2024, Elsevier, (b) the global corrosion inhibitors market and forecast 2023–2034 (USD billion), (c) corrosion inhibitor market share by region in 2022, compiled from https://www.precedenceresearch.com.

Fig. 11a and b show the development of corrosion inhibitors and its global market over time in a chronological manner. Organic corrosion inhibitors dominated the market in 2020 and accounted for over 60% of the total market share. The growing popularity of organic inhibitors, such as amines and imidazolines, is driven by their versatility and effectiveness in various applications. The organic sub-segment had the greatest corrosion inhibitors market share three years ago. The corrosion inhibitors market value for this sub-segment was 4975.5 million back then. The organic sub-segment is expected to have a CAGR of 4.13% for the period that this report covers. The inorganic corrosion inhibitors market growth rate is expected to be 4.5% for this period. Green corrosion inhibitors is projected to witness the highest CAGR during the forecast period, as there is an increasing demand for environmentally friendly and sustainable corrosion protection solutions.

In 2020, North America emerged as the dominant regional market, capturing over 34% of the global market share, primarily bolstered by its well-established industrial base and strict regulatory frameworks in the United States and Canada (Fig. 11c). On the other hand, Asia-Pacific stands out as the fastest-growing regional market, exhibiting a remarkable CAGR of over 8% throughout the forecast period. Meanwhile, Europe ranks as the third-largest regional market, with notable market presence in countries. Moreover, the evolving regulatory landscape and mounting environmental concerns are ushering in a shift towards the adoption of less toxic and environmentally friendly corrosion inhibitor products. This trend highlights the industry's continuous evolution towards sustainable and eco-conscious practices in response to global environmental challenges.

## 7. SWOT analysis

**Fig. 12:**
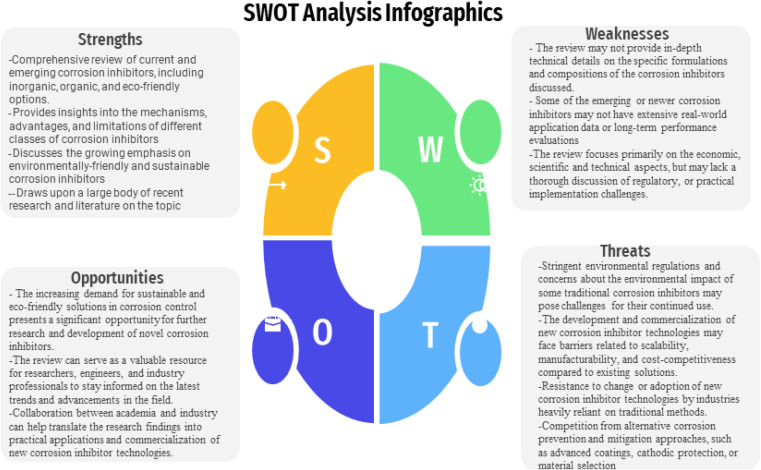
SWOT analysis.

The review highlights the potential of synergistic effects between mixed corrosion inhibitors, particularly organic/organic systems, as a viable and advantageous choice for applications requiring robust corrosion inhibition performance in challenging processing environments. These mixed inhibitors offer the benefits of low environmental risk and high efficiency, positioning them as a preferred alternative to single-system inhibitors. While the review article provides a comprehensive technical overview, it may fall short in offering detailed formulation-level insights, limiting the ability of readers to fully understand the nuances and optimization potential of these inhibitor systems. Additionally, the limited coverage of real-world application data and long-term performance evaluation could undermine the confidence of end-users in adopting the discussed inhibitor technologies. Furthermore, the review's narrow focus on the technical aspects may overlook critical factors such as regulatory, safety, and practical implementation challenges, which are crucial for the successful deployment of corrosion control solutions in the real world. This lack of a more holistic perspective could hinder the review's ability to provide a comprehensive roadmap for the widespread adoption of the discussed inhibitor technologies. Opportunities for expanding the review's scope include incorporating more diverse global perspectives, exploring hybrid and synergistic inhibitor systems, integrating life cycle assessment and sustainability analysis, establishing industry partnerships, and broadening the coverage to include alternative corrosion mitigation techniques. Addressing these areas could enhance the review's relevance and applicability across different regions and industries. Potential threats to the widespread adoption of corrosion inhibitors include regulatory and environmental concerns, technological advancements in competing corrosion control methods, economic and market fluctuations, resistance to change in established industries, and the lack of standardized testing and evaluation protocols. Addressing these challenges will be crucial for the successful implementation of effective and sustainable corrosion inhibition strategies. Fig. 12 demonstrated the summary of SWOT analysis.

## 8. Conclusion and prospects

This review article provides a comprehensive and in-depth analysis of corrosion inhibitors, with a focus on both inorganic and organic inhibitors, as well as ecofriendly and biological macromolecules. The review highlights the inhibition mechanisms, with a particular emphasis on the efficiency of organic compounds due to the presence of heteroatoms and conjugated π electron systems. The review also presents case studies and investigations of corrosion inhibition, showcasing the performance and potential application of various inhibitors. One significant aspect that this review addresses is the growing trend of seeking eco-friendly alternative inhibitors derived from natural resources. The review provides a comparative evaluation of the environmentally friendly biopolymer inhibitors, considering their efficacy, compatibility, and sustainability. Furthermore, the evaluation of corrosion inhibitors is discussed, encompassing various analytical techniques such as weight loss, electrochemical, and surface analysis tools. This comprehensive evaluation enhances our understanding of inhibitor behaviors and mechanisms; however, there are some gaps that need to be filled, including:

(1) Optimizing existing inhibitors: researchers should focus on enhancing the effectiveness and stability of currently available organic, inorganic, and green corrosion inhibitors. This can be achieved through strategic molecular design, the incorporation of synergistic additives, and the development of inhibitor-based coatings and composite systems. Particular attention should be given to improving the long-term durability and reliable performance of inhibitor films under challenging environmental conditions.

(2) Synergistic inhibition in extreme environments: investigating the performance of mixed/hybrid/and composite inhibitors under harsh and demanding conditions, such as high temperatures, aggressive chemical environments, or elevated mechanical stresses, can unlock new avenues for corrosion control. Demonstrating the synergistic inhibition capabilities in these extreme scenarios can expand the applicability of these systems to challenging industrial settings.

(3) Enhanced mechanistic insights: delving deeper into the mechanistic aspects of synergistic inhibition using mixed inhibitors can yield valuable scientific contributions. Elucidating the precise interactions between the inhibitors, their influence on passive film formation, and the interplay between anodic and cathodic processes can provide fundamental knowledge to guide the design of more effective corrosion mitigation strategies.

(4) Multilayered inhibitor architectures: building upon the concept of synergistic inhibition, the prospect of designing multilayered inhibitor architectures on metal surfaces presents an intriguing direction. By strategically arranging different inhibitors in tailored sequences, it may be possible to create hierarchical protective systems with enhanced durability and self-healing capabilities.

(5) Bioinspired and biomimetic inhibitors: the pursuit of green and eco-friendly corrosion inhibitors has led researchers to explore bioinspired and biomimetic approaches. Drawing inspiration from naturally occurring processes and structures, scientists are investigating the development of inhibitors that mimic the self-healing, self-cleaning, or anti-fouling properties found in biological systems. For instance, the study of marine organisms and their inherent resistance to corrosion has the potential to yield novel biomimetic inhibitor formulations with enhanced performance and environmental compatibility.

(6) Multifunctional and responsive smart inhibitors: the next generation of corrosion inhibitors is expected to exhibit multifunctional and smart capabilities, addressing not only corrosion prevention but also other surface-related challenges, such as antifouling, anti-icing, or self-healing properties. These responsive and adaptive inhibitors would be able to sense and respond to changes in the environment, automatically adjusting their protective functions to maintain optimal performance under varying conditions.

(7) Application of computational modeling, machine learning, and artificial intelligence that significantly accelerate the discovery, optimization, and deployment of more efficient corrosion inhibitors.

(8) Scaling up green inhibitor production: to meet the growing demand for eco-friendly corrosion inhibitors, researchers and industry should collaborate to develop scalable extraction, purification, and formulation processes for naturally occurring inhibitor compounds derived from plant extracts, marine organisms, or other renewable sources. Simulation and modeling tools can play a crucial role in optimizing the production parameters and enhancing the industrial-scale viability of these green inhibitors.

(9) Sustainable production and life cycle assessment: as the focus on environmental sustainability intensifies, the development of corrosion inhibitors will need to be accompanied by sustainable production processes and comprehensive life cycle assessments. This will involve the use of renewable, biodegradable, and non-toxic raw materials, the optimization of manufacturing methods to minimize waste and energy consumption, and the implementation of circular economy principles to enable the reuse, recycling, or safe disposal of inhibitor-containing products.

In conclusion, this review article not only provides a comprehensive analysis of corrosion inhibitors but also highlights the importance of adopting environmentally friendly alternatives. It offers valuable insights and future perspectives for researchers and industrial sectors, ultimately helping to build effective and sustainable corrosion control solutions.

## Data availability

The data analyzed in this review article are from previously published studies. The specific datasets and sources are cited throughout the manuscript and listed in the reference section. Readers can access the underlying data from the original published sources as cited. The authors confirm that they did not have any special access privileges to these datasets.

## Conflicts of interest

There are no conflicts to declare.

## Supplementary Material

RA-014-D4RA05662K-s001
