# Supplementary material for: Current and emerging trends of inorganic, organic and eco-friendly corrosion inhibitors
Source: RSC Adv. 2024 Oct 8;14(43):31877–920. doi: 10.1039/d4ra05662k (PMC11460216; doi:10.1039/d4ra05662k)
Supplement: RA-014-D4RA05662K-s001 [file RA-014-D4RA05662K-s001.pdf]

Supplementary Information (SI) for RSC Advances.
This journal is © The Royal Society of Chemistry 2024

Current and Emerging Trends of Inorganic, Organic and Eco-Friendly Corrosion Inhibitors

Mahmoud A. Ahmed, [a,b]* Sherif Amine [b] , Ashraf A. Mohamed [a]

[a] Chemistry department, Faculty of Science, Ain Shams University, Cairo-11566, Egypt

[b] Veolia Water Technologies, Cairo 11835, Egypt

*** Corresponding authors**
Mahmoud A. Ahmed (E-mail address: mahmoudmahmoud_p@sci.asu.edu.eg)

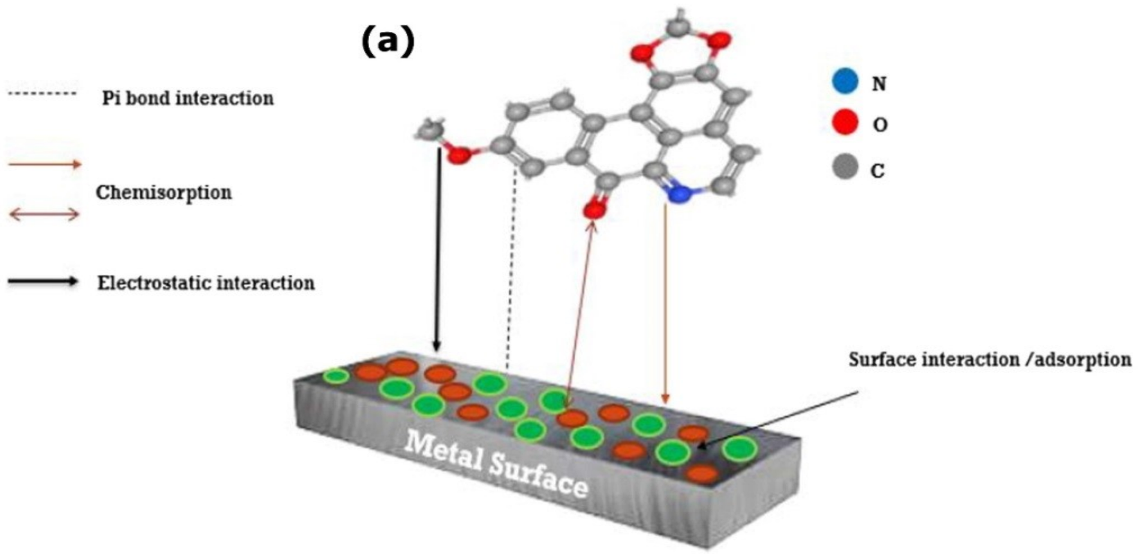

Figure S1. (a) Physicochemical mechanisms Reprinted with the permission of ref [36], copyright 2024, Elsevier. [36].

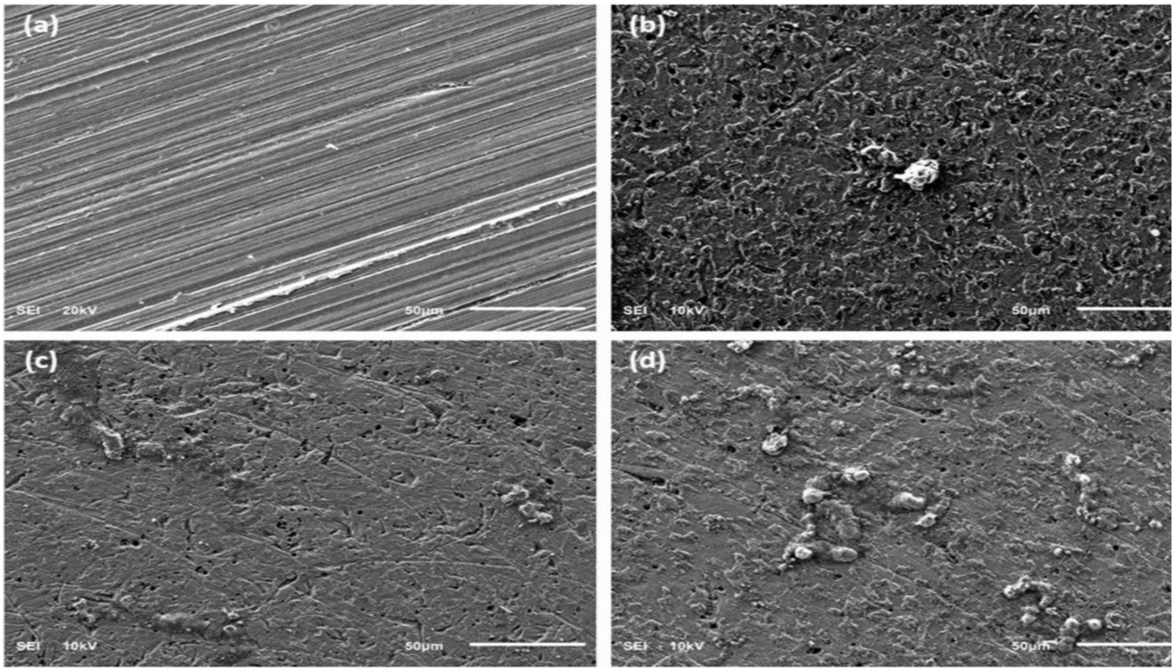

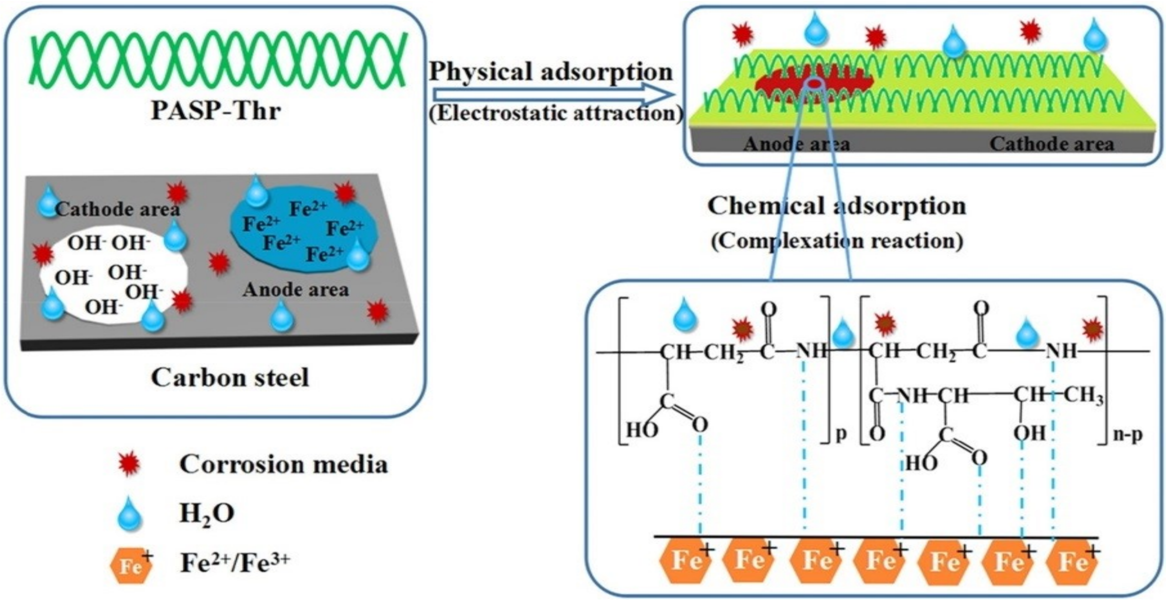

Figure S2. SEM of mild steel in 3% NaCl solution (a) before immersion, after immersion (b) without inhibitor, (c) with 2.0 g/L PASP (d) with 2.0 g/L PASP and 0.01 g/L Zn, Reprinted with the permission of ref [45], copyright 2024, Elsevier. , (e) The corrosion inhibition process of PASP-Thr for carbon steel in simulated cooling water[46].

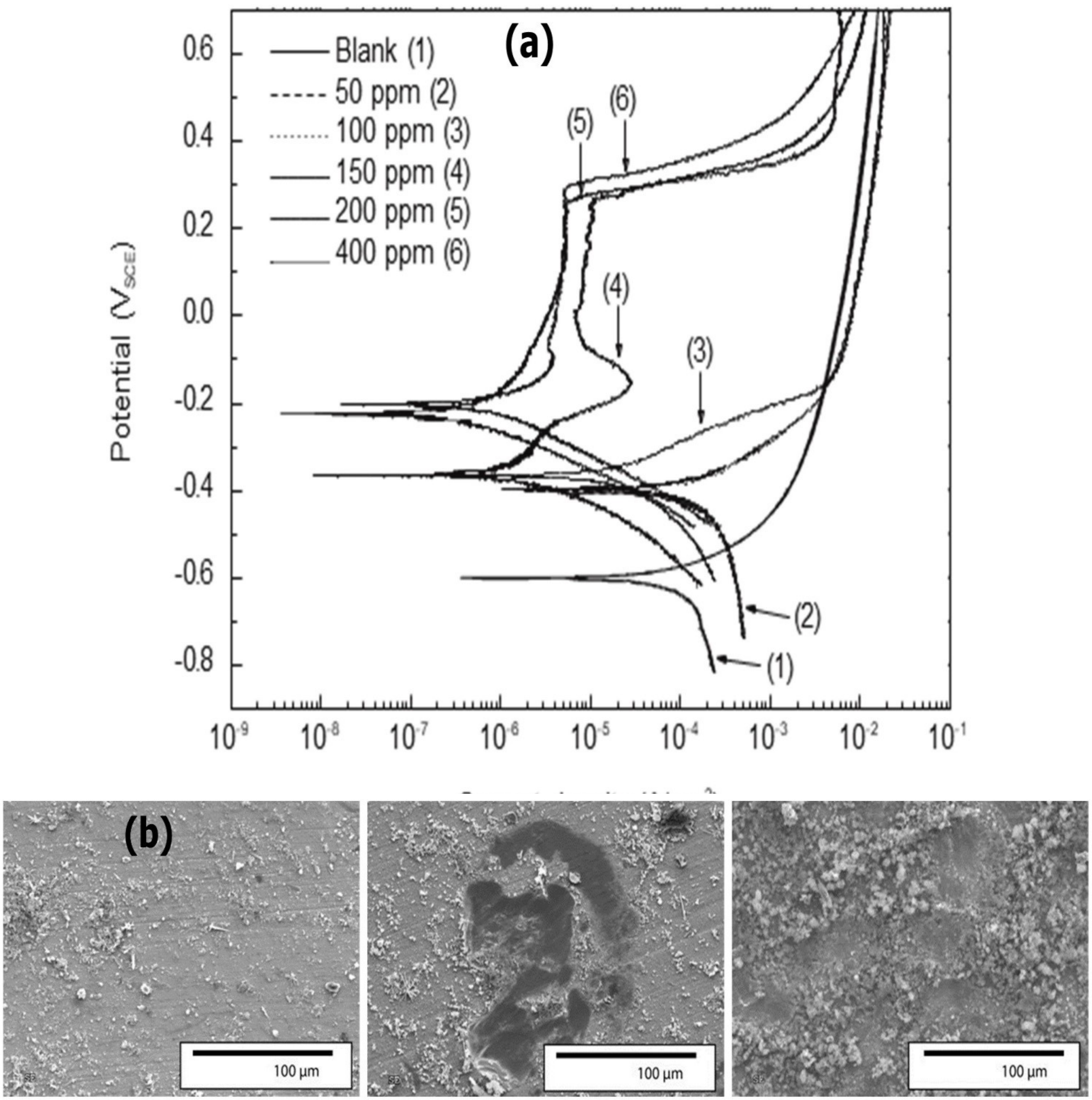

Figure S3(a) Polarization curves of carbon steel at various nitrite levels, SEM image, (b) in 200 ppm nitrite solution, (c) 100 ppm nitrite solution, and (d) 50 ppm nitrite solution. Reprinted with the permission of ref [57], copyright 2024, Elsevier. .

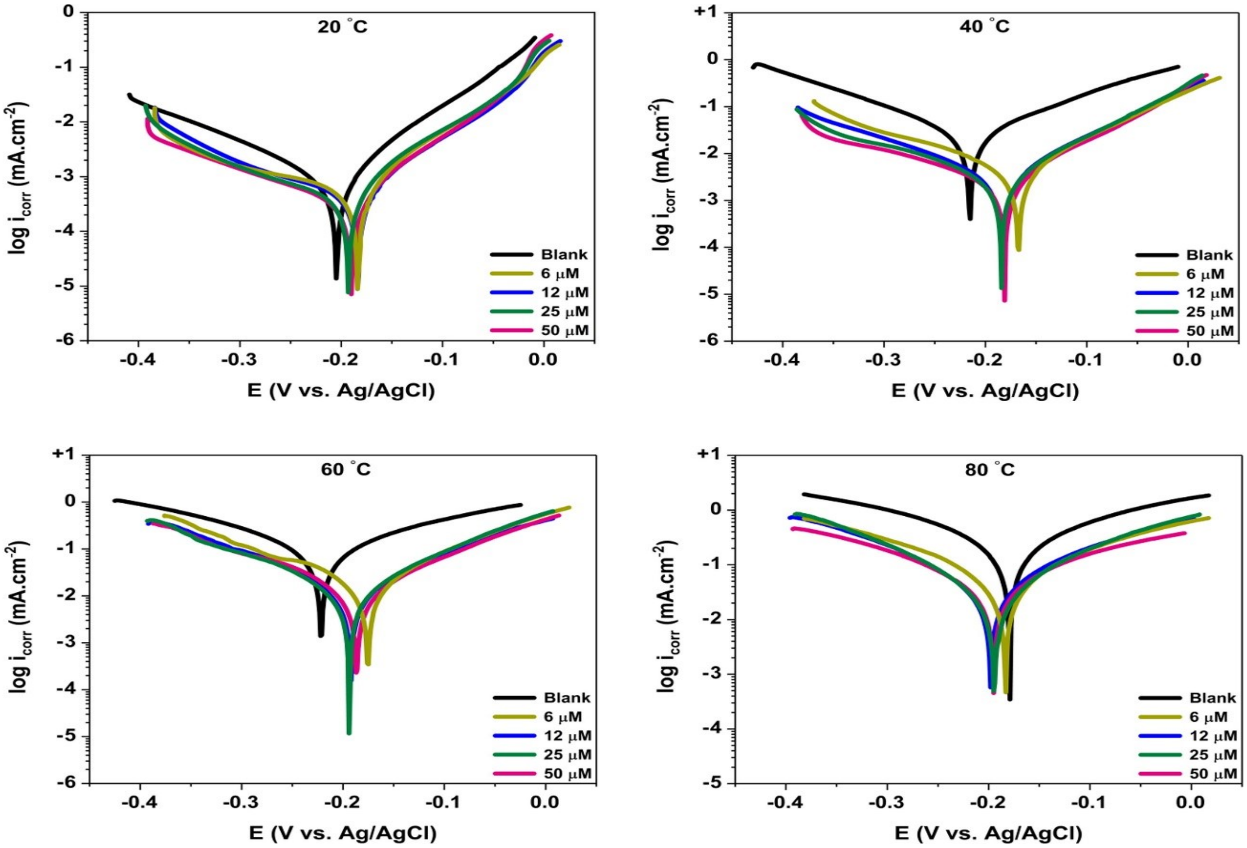

Figure S4. Plots of PDP for blank and SDCI solutions at 303 K, 323 K, 343 K, and 363 K, Reprinted with the permission of ref [331], copyright 2024, Elsevier..

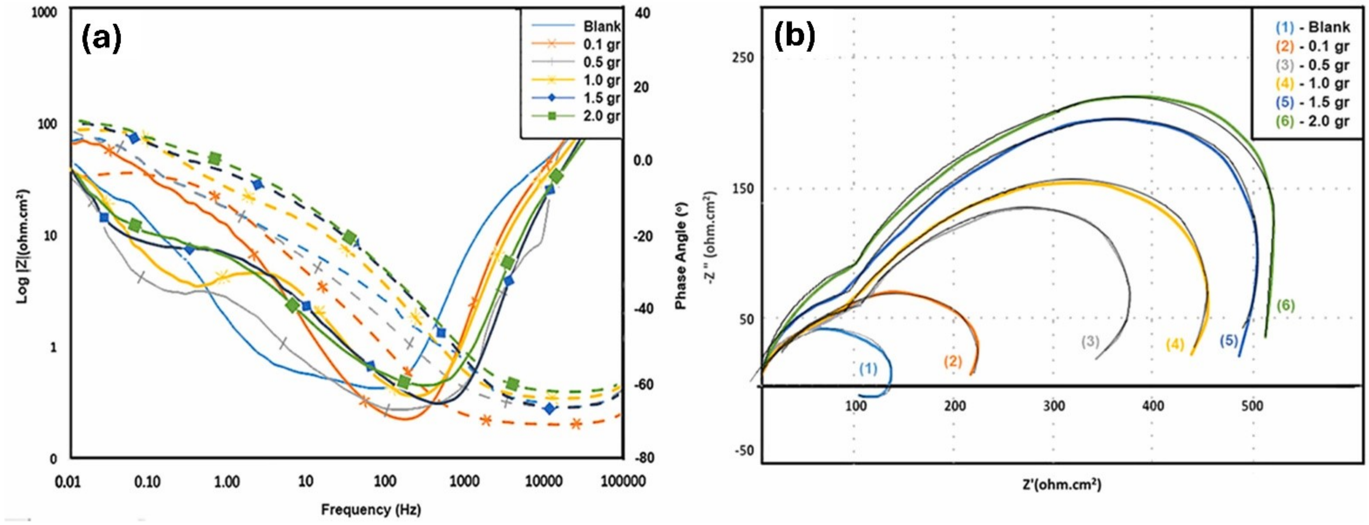

Figure S5. EIS spectra of mild steel in the presence and absence of PESA-Zn^{+2} inhibitor (a) Bode/Phase angle plot (b) Nyquist plot, Reprinted with the permission of ref [338], copyright 2024, Elsevier.

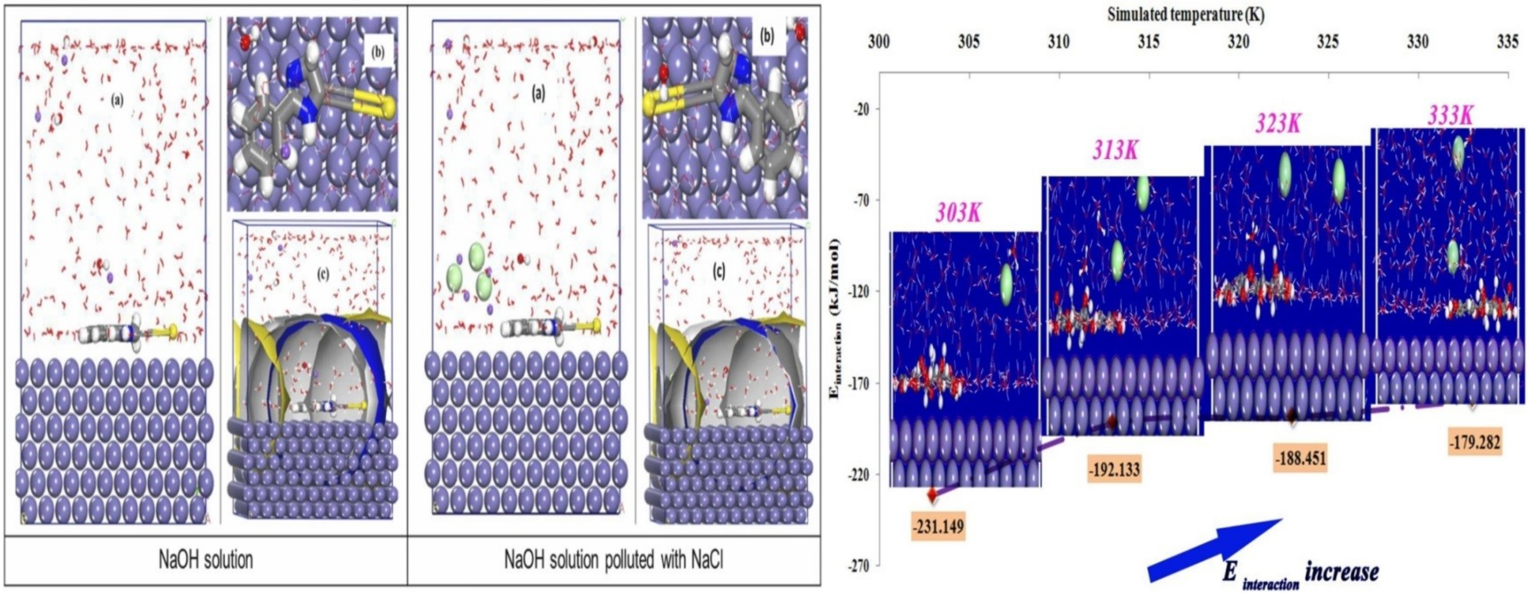

Figure S6. (a) PTT/Fe(110) adsorption system: (a) Side view; (b) Top view; and (c) Coverage density views of the adsorption of PTT inhibitor molecules on the Fe surface in NaOH solution[355] , (d) Variation of interaction energy with the temperature (303, 313, 323 and 333 K) for adsorbed LM molecule on Fe(110),. Reprinted with the permission of ref [351], copyright 2024, Elsevier.
